# Antibody Binding to Peptide Conjugated Gold Nanoparticles Preferring Dense Antigen Coating with Small Deflection Angle

**DOI:** 10.1021/acsnanoscienceau.6c00049

**Published:** 2026-07-07

**Authors:** Xinyu Ning, Yuan Guo, Dejian Zhou

**Affiliations:** † School of Chemistry, Astbury Centre for Structural Molecular Biology and Bragg Centre for Materials Research, 4468 University of Leeds, Leeds LS2 9JT, U.K.; ‡ School of Food Science and Nutrition and Astbury Centre for Structural Molecular Biology, University of Leeds, Leeds LS2 9JT, U.K.

**Keywords:** Antibody−antigen interaction, Gold nanoparticle, Binding affinity, Fluorescence quenching, Dynamic light scattering, Antigen presentation, His-tag

## Abstract

Antibody–antigen interactions are fundamental to immune defenses against pathogen infection, where antibody binding affinity and mode control functional outcomes. While literature show optimal IgG binding on flat surfaces occurring at relatively large interantigen distances (*d* ≈ 10–16 nm), how nanoscale curvature affects IgG binding remains unclear. Here, we present a model antigen–nanoparticle system by displaying varying densities of His_6_ peptides on gold nanoparticles (GNPs). Using GNPs’ nanoscale size and strong fluorescence-quenching capability, we probe their binding with anti-His antibody via dynamic light scattering (DLS) and GNP-based fluorescence quenching assays. We find that DLS detects only stable, chelative complexes, whereas the GNP quenching assay captures both stable and dynamic interactions and quantifies their affinities. We show that antibody binding is highly sensitive to antigen density: reducing His_6_ density from 10% to 2% (*d* increasing from ∼3.3 to ∼6.3 nm) weakens affinity by over 120-fold. For antigen–GNP conjugates with *d* values within the antibody’s accessible spacing range, the antigen deflection angle θ dominates binding behavior: θ ≤ 16° favors strong chelative binding, whereas θ ≥ 37° permits mainly weak monovalent binding.

## Graphical abstract

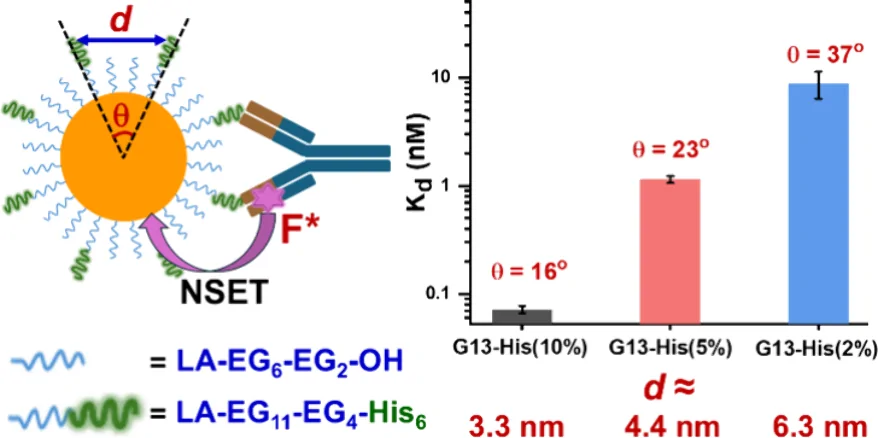

Antibody–antigen interactions are central to immune defense, allowing the host to recognize, neutralize, and eliminate pathogens.^1^ IgG, the most abundant plasma antibody, has a Y-shaped structure with two antigen-binding Fab arms and an Fc region connected by a flexible hinge.^2^ IgG can chelate 2 epitopes on a target via its 2 Fabs, to enhance affinity over monovalent binding. Once bound, the Fc domain engages Fc receptors on immune cells or activates the complement system to drive pathogen clearance.^3,4^ Because many pathogens display repetitive antigen patterns, strong chelating IgG binding results in dense IgG coating, promoting Fc receptor clustering on immune cells to trigger immune activation.^3,5,6^ However, some pathogens have evolved strategies to evade immune recognition. For example, influenza viruses employ antigenic drift and shift, continually changing their epitopes on spike proteins such as hemagglutinin (HA) and neuraminidase (NA) to prevent recognition by existing host antibodies.^7^ HIV-1 poses an even greater challenge: it mutates rapidly and its trimeric gp160 spikes are heavily glycosylated, shielding conserved functional regions from antibody recognition.^8^ Although broad neutralizing antibodies (bnAbs) can penetrate this glycan shield using long binding loops and bind multivalently to conserved spike regions,^9,10^ bnAbs are very rare and take years to develop in infected individuals.^11^ Antigen presentation on pathogens is key to dictate antibody binding affinity and clustering. For instance, an ∼100-nm spherical influenza virion typically displays 300–400 HA and 40–50 NA spikes, with NA spikes often clustered on one side of the virion to form high-density targets that support strong bivalent IgG binding for efficient neutralization.^12,13^ In contrast, HIV-1 virions have only 7–14 gp160 spikes randomly spread over a similar size particle.^14,15^ This extremely sparse gp160 spikes (resulting in large interspike distances) together with dense glycan shields effectively prevent strong IgG binding from both intraspike and interspike cross-linking,^15^ allowing the HIV to evade immune surveillance.

Chelative binding is essential to maximize antibody–antigen binding affinity. This requires matching the interantigen distance (*d*) with that of the 2 Fab antigen-binding sites, typically ranging from 11.7 to 13.4 nm for various IgG antibodies.^16^ Using a coiled-coil structure to adjust inter-His-tag antigen distances, Jendroszek et al. revealed that *d* = 13 nm gave the maximal IgG affinity (∼30-fold that of monovalent binding).^17^ Similarly, the Fan group used antigens presented on DNA origami to capture IgG binding and revealed an optimal *d* value of ∼10 nm.^18^ IgG’s spatial freedom is governed by its angular flexibility, including the highly variable internal elbow angle within each Fab,^19^ and the interarm angle due to hinge flexibility.^16^ This feature enables IgGs to tolerate broad distances, from ∼ 3 to ∼ 17 nm, on nanopatterned antigen surfaces, although their IgG affinities are notably weaker at both short (3–7 nm) and long (∼17 nm) *d* values.^20^ Besides, IgG affinity enhancement depends strongly on antigen density and monovalent affinity.^21^ These studies revealed that a *d* value of ∼10–16 nm is optimal for IgG binding on flat surfaces, depending on IgG subclass and assay methods.

We hypothesize that the optimal *d* for strong IgG binding may differ for antigens presented on curved nanoparticles from those on flat surfaces. As shown schematically in Figure 1A, for antigens displayed on flat surfaces, the optimal *d* for IgG chelative binding is governed by its natural interbinding site distance (i.e., 11.7–13.4 nm),^16^ matching well to what reported above. For antigens displayed on nanoparticle surfaces, both *d* and orientation (deflection angle, θ) may influence IgG binding. The epitopes must be aligned properly to match IgG’s two binding sites, as excessively large θ can hinder chelation even if *d* is optimal (Figure 1B). This key aspect of antibody binding to antigen-conjugated nanoparticles remains largely unexplored. This is especially relevant for complex structures such as viruses, where steric constraints can hinder IgG chelative binding, thereby reducing binding affinity and neutralization potency.^22^

**Figure 1:**
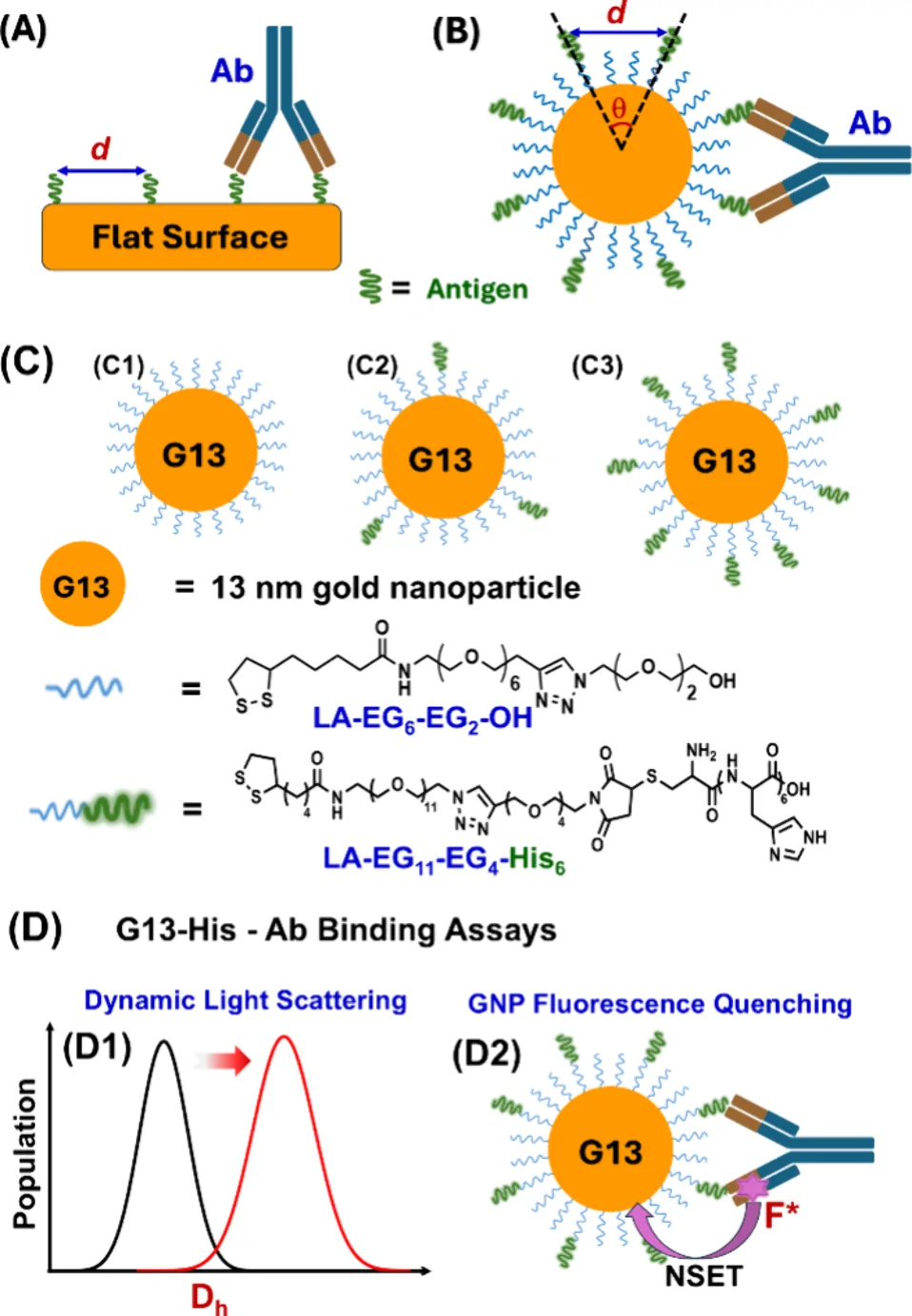
(A, B) Schematic comparison of an IgG antibody binding to antigen presented on a flat (A) and curved nanoparticle (B) surfaces. On a flat surface, *d* is the main factor controlling IgG chelative binding, whereas on nanoparticle surface, both *d* and θ may play an important role in controlling Abs binding. (C) Schematic structures of G13-OH (C1, negative control) and G13-His conjugates (C2 and C3) with varying His_6_ densities. The chemical structures of LA-EG_6_-EG_2_–OH spacer and LA-EG_11_-EG_4_-His_6_ ligands are depicted beneath. (D) Study G13-His-Abs interactions by (D1) following G13-His hydrodynamic size changes upon Abs binding by dynamic light scattering, and (D2) a GNP fluorescence quenching assay: binding of Ab to G13-His brings its Att643 labels close to G13, leading to efficient quenching of Atto643 fluorescence by G13 via a nano surface energy transfer (NSET) mechanism.

To address this knowledge gap, we report our preliminary study by coating an ∼13-nm gold nanoparticle (G13) with varying densities of a model His_6_ peptide antigen to probe how antigen presentation affects binding to an anti-His antibody (HisAb). The His_6_ peptide, commonly used as an affinity tag for protein purification, was selected due to the availability of commercial HisAbs. G13 was coated with a lipoic acid–oligo­(ethylene glycol)-His_6_ ligand (LA-EG_11_- EG_4_-His_6_), diluted to varying degrees with an inert spacer ligand, LA-EG_6_-EG_2_–OH (Figure 1C). Both ligands use the same LA domain to anchor on G13 surface by forming two strong Au–S bonds, they should have the same G13 anchoring ability, allowing us to control the His_6_ density (*d*) and orientation (θ) on the G13 surface by varying the His_6_ ligand content in ligand mixture used in incubation (Figure 1C).^23,25^ Interactions between G13–His_6_ conjugates (G13-His­(*x*%), where *x*% stands for the G13 surface His_6_-ligand content (as a percentage), which is assumed to be the same as that of the incubation solution) and HisAbs were studied by following the hydrodynamic diameter (*D*_h_) of G13-His–HisAb complexes via dynamic light scattering (DLS; Figure 1D1) and by a gold nanoparticle (GNP)-based fluorescence quenching assay using Atto643-labeled HisAbs (HisAb-Atto643; Figure 1D2). We have shown previously that the GNP-quenching assay is highly robust, offering quantitative binding data, e.g., equilibrium dissociation constant (*K*_d_) and thermodynamics (Δ*H*, Δ*S*, Δ*G*) for multivalent interactions between glycan-GNPs and lectins.^23–25^ Here, this assay was employed to probe how antigen presentation, *d* and θ, control antibody binding to antigen–GNP conjugates.

Each LA-EG_11_-EG_4_-His_6_ ligand was designed to contain 3 functional domains:^23,24^ a LA anchoring domain to provide robust anchoring onto the G13 surface by forming two stable Au–S bonds; a flexible, hydrophilic EG_n_ linker to afford high water solubility, resisting nonspecific interactions,^26,27^ and extend the terminal His_6_ away from G13 surface to improve accessibility; and a His_6_ antigen for specific Abs binding. LA-EG_11_-EG_4_-His_6_ was synthesized in two-steps (see Scheme S1 in the Supporting Information (SI)). First, LA-EG_11_-N_3_ (synthesized previously)^28,29^ was coupled to HC≡C-EG_4_-maleimide via Cu­(I)-catalyzed click reaction to give LA-EG_11_-EG_4_-Mal, which was then conjugated to the commercial Cys-His_6_ peptide via Michael addition. After purification by P2 Biogel size exclusion chromatography,^23,24^ the desired LA-EG_11_-EG_4_-His_6_ ligand was obtained and confirmed by mass spectroscopy (see Figures S1 and S2 in the SI). Besides, the LA-EG_6_-EG_2_–OH spacer ligand was also synthesized by Cu­(I)-catalyzed click reaction between LA-EG_6_-C≡CH and N_3_-EG_2_–OH as reported previously. Its chemical structure was confirmed by MS and NMR (see Figures S3–S5 in the SI).^24^

Citrate-stabilized G13 was prepared by citrate reduction of H­[AuCl_4_], as reported previously (see Figure S6 in the SI).^23,30^ They were incubated with mixed LA-EG_11_-EG_4_-His_6_ and LA-EG_6_-EG_2_–OH ligands, with the His_6_-ligand content% varying from 0% (refers as G13-OH, G13 coated with 100% LA-EG_6_-EG_2_–OH ligand, serving as a negative control) to 15%. The total LA-ligand: G13 molar ratio (LGMR) was fixed at 3000 as our calculation indicates that this ratio can fully saturate G13 surface with a robust monolayer of the LA-ligands (see page S8 in the SI).^23^ Both ligands anchor on G13 surface via the same LA domain by forming 2 stable Au–S bonds, hence exhibiting the same anchoring ability, allowing us to tune G13 surface His_6_ content% by varying the His_6_-ligand content% in ligand mixtures used in incubation.^23,25^ Compared to parent citrate-coated G13, all G13-His conjugates exhibited a small ∼ 4 nm redshift in SPR absorption, due to the increased local refractive index after LA-ligand coating (see Figure S7 in the SI).^23^ All G13-His conjugates, except G13-His(15%) which aggregated and thus was excluded from further binding studies, were stable and monodisperse in PBS (pH 7.4), with *D*_h_ values consistent with single G13 cores coated by a monolayer of the corresponding LA-ligands. Their *D*_h_ values increased gradually with the increasing LA-EG_11_-EG_4_-His_6_ content, rising from ∼18.5 nm for G13-OH to ∼22.9 nm for G13-His(10%), consistent with the coating of higher amounts of the longer LA-EG_11_-EG_4_-His_6_ ligand (see Figure S8 and Table S1 in the SI). The terminal groups in both ligands (His_6_ and OH) are small and extend away from the G13 surface via a flexible and long EG_*n*_ linker, thus they should not cause steric hindrance during G13 anchoring. Ligand loading on each G13 should be governed solely by the LA anchor group and be constant for conjugates prepared at the same LGMR. Accordingly, the total number of LA-EG*_n_* ligands on each G13 was assumed to be ∼1960, the same as this batch of G13 coated with a LA-EG_4_-psDiMan ligand under an identical LGMR of 3000.^23^

Using the mean *D*_h_ value (peak width, ±^1^/_2_ W, not considered) and His_6_ valency (*N*, the number of His_6_ ligand on each G13 surface, error bars considered) of each G13-His conjugate, the average inter-His_6_ distance (*d*), antigen footprint (*k*), and deflection angle (θ) were calculated using the method first reported by the Mirkin group,^31^ and the data are summarized in Table 1. Importantly, the *d* values for all G13-His(1–10%) conjugates fall within the reported IgG accessible range (3–17 nm),^20^ while their *d* and θ are enlarged systematically with the decreasing His_6_ density, allowing us to probe how these parameters govern antibody binding.

**Table 1:** Summary of Key His_6_ Presentation Parameters of G13-His Conjugates of Varying His_6_ Densities

His_6_ density (%)	*D*_h_ ^a^ (nm)	*N*	*k* (nm^2^)	θ (deg)	*d* (nm)
0	18.5 ± 3.8	–	–	–	–
1	18.5 ± 3.8	20 ± 2	54.8 ± 5.2	51.7 ± 2.5	8.4 ± 0.4
2	19.6 ± 4.4	39 ± 4	30.7 ± 2.9	36.6 ± 1.7	6.3 ± 0.3
5	21.6 ± 5.0	98 ± 9	14.9 ± 1.4	23.1 ± 1.1	4.4 ± 0.2
10	22.9 ± 4.4	196 ± 19	8.4 ± 0.8	16.4 ± 0.8	3.3 ± 0.2

a *D*_h_ is given as mean ± ^1^/_2_ W, half the full-width at half maximum of Gaussian fit.

The interactions between G13-His and HisAbs were first studied by DLS. HisAbs alone gave a *D*_h_ of ∼11.6 nm in PBS (Figure S9A). Representative *D*_h_ histograms of G13-His­(5% and 10%, 2 nM each) without and with 64 nM HisAb are shown in Figures 2A–D, while those for all other conditions were given in the SI (Figures S9–S13). All G13-His(0–10%) + HisAb samples showed single, narrowly distributed species with *D*_h_ values ranging from ∼19 (G13-His(0%)) to ∼32 (G13-His(10%)) nm and no larger aggregates. This conclusively rules out the formation of stable, cross-linked G13-His–HisAb assemblies where each HisAb binds monovalently to two separate G13-His particles. The average *D*_h_ (mean ± ^1^/_2_ *W*) and *D*_h_ change (Δ*D*_h_) values, as a function of the HisAb:G13 molar ratio, are given in Figure 2E and 2F, respectively.

**Figure 2:**
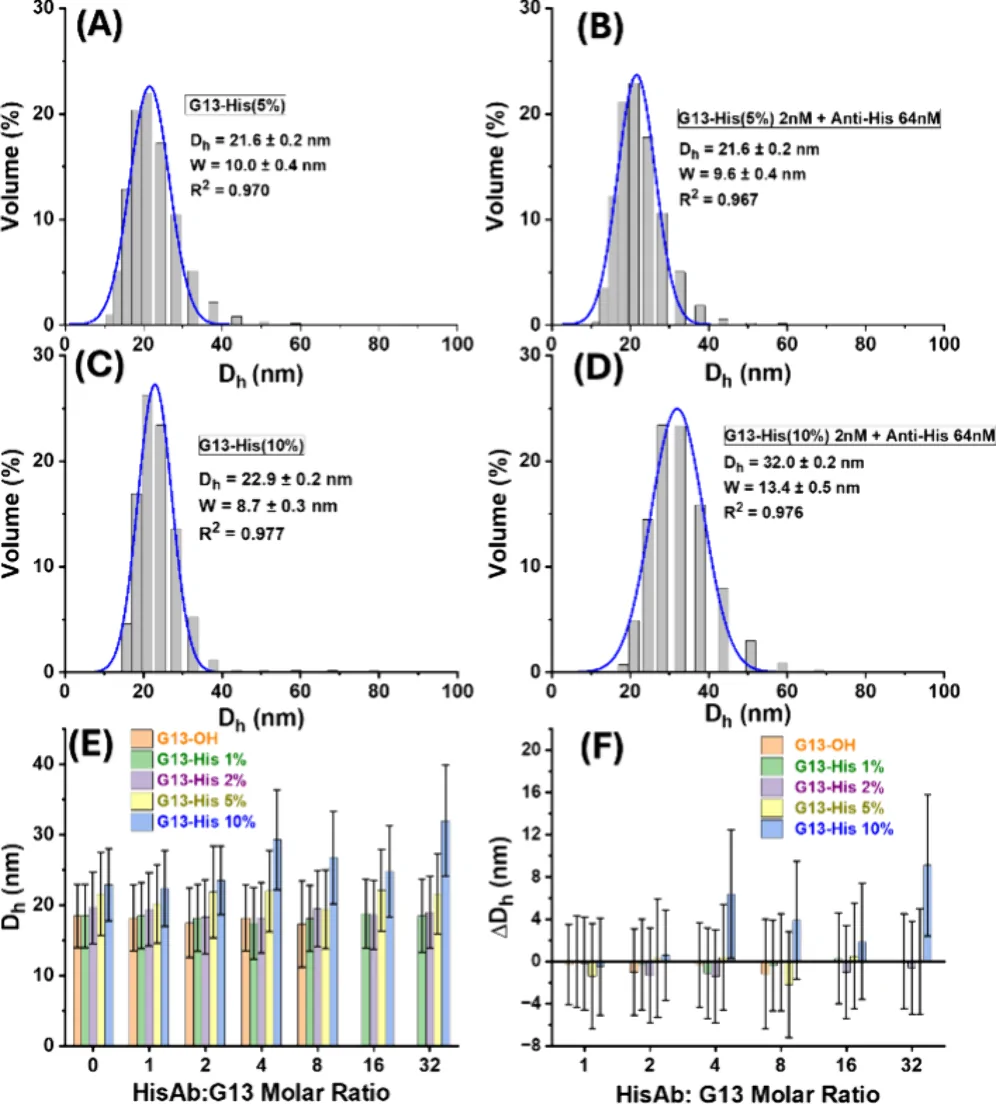
(A–D) *D*_h_ histograms of 2 nM G13-His­(5% (A, B) and 10% (C, D)) without (A, C) and with (B, D) 64 nM of HisAb. (E) Comparison of *D*_h_ (mean ± ^1^/_2_ W, half the full-width at half-maximum of Gaussian fit) and (F) *D*_h_ change (Δ*D*_h_, after subtraction of the *D*_h_ of the G13-His conjugate alone) for G13-His(0–10%) binding with HisAbs at varying HisAb:G13 molar ratios.

No apparent *D*_h_ increases were observed for G13-His conjugates with His_6_ density of ≤5% (G13-His(≤5%)), indicating no detectable HisAb binding under such conditions. In contrast, G13-His(10%) showed clear *D*_h_ increases at HisAb:G13 molar ratios ≥ 4 (Figure 2F), with a maximal Δ*D*_h_ of ∼10 nm obtained at a ratio of 32. This is consistent with the formation of stable G13-His(10%)–HisAb complexes, where HisAbs chelate to a single G13-His particle and forming a monolayer of antibodies at saturation. These results highlight the critical role of antigen density on G13 surface in enabling strong HisAb binding. Although the average *d* values for all G13-His(1–10%) conjugates (3.3–8.4 nm; Table 1) fall within the accessible range of IgG’s 2 Fabs on flat surfaces (∼3–17 nm),^20^ the large θ imposed by the curved G13 surface may limit epitope reorganization, preventing the spatial and orientational alignment needed for strong chelative antibody binding. Here, only G13-His_6_(10%) exhibits a θ value of ∼16° that is small enough to permit strong chelative antibody binding. Besides, the flexible EG_11_-EG_4_ linker used to display the His_6_ antigen may impose entropic penalties during chelation, resulting in weaker affinity than more rigidly presented antigens.^18,20^ Hence, a higher antigen density (i.e., smaller *d*) is required to increase the chances of statistical rebinding, maximizing their affinity. Here, we find that the optimal *d* for strong antibody binding decreases substantially, from ∼10–16 nm on flat surfaces^18,20^ to ∼3.3 nm for G13-His(10%) on the curved G13 surface. This result indicates that antibodies require much higher antigen densities on curved nanoparticles than on flat surfaces to achieve strong binding.

In fact, many viruses display spike proteins on spherical or near-spherical enveloped structures with diameters of ∼100–120 nm. Their interspike distances are typically within the accessible range of IgG’s two Fab arms. For example, the HA spikes on influenza viruses are spaced at ∼11 nm apart.^12^ This translates into a *θ* value of ∼13° for the HA spikes (calculated using an inter-HA distance of 11 nm on a 100 nm spherical virion via eq S3, SI), which is comparable to ∼16° in G13-His(10%). For nonspherical (pleomorphic) or filamentous viruses,^32^ such as Ebola and some influenza strains, spike proteins at high-curvature regions can exhibit large *θ* values, where geometric constraints may impede Abs forming strong chelative binding, limiting their viral neutralization potency.

The binding of HisAbs to G13-His(0–10%) was studied by a GNP fluorescence quenching assay recently developed in our lab.^23–25^ GNPs efficiently quench a range of fluorophores via a nanosurface energy transfer (NSET) mechanism,^33,34^ where the quenching efficiency (QE) is inversely dependent on the fourth power of the fluorophore-GNP distance.^33,34^ Since significant quenching occurs only over short distances (<50 nm), this must come from G13-His–HisAbs interactions at low concentrations (sub-10 nM here). In this regard, HisAb was labeled randomly with Atto643-NHS ester, giving an average dye label ratio of ∼ 2.3 (denoted as HisAb-Atto643). Varying concentrations (from 0.10 to 6.4 nM) of HisAb-Atto643 were mixed with 1 mol equiv of G13-His conjugates (i.e., HisAb-Atto643:G13-His conjugate molar ratio fixed at 1:1) in PBS containing large excess bovine serum albumin (BSA, 1 mg/mL),^23,25^ a nontarget protein to block nonspecific interactions and minimize nonspecific adsorptions on the surfaces, which can be a major source of experimental error at sub-10 nM concentration.^23,24,35^ Fluorescence spectra (650–800 nm) were recorded under a fixed λ_EX_ of 630 nm, where G13 has minimal absorption to minimize the interference of G13’s inner filter effect in fluorescence measurement.^23,25^ Fluorescence spectra of HisAb-Atto643 only samples at these concentrations were also recorded and used to calculate QE at each concentration (*C*) via eq 1:^23,25^

1 $$\mathrm{QE}\;(\%)=\frac{{\mathrm{IF}}_{0}-\mathrm{IF}}{{\mathrm{IF}}_{0}}\times 100$$

where IF_0_ and IF are the integrated fluorescence of HisAb-Atto643 in the absence and presence of G13-His conjugate, respectively. The apparent *K*_d_ was derived from fitting the QE–*C* plots with the Hill’s equation (eq 2):

2 $$\mathrm{QE}=\frac{{\mathrm{QE}}_{\max}\times C^{n}}{K_{d}^{n}+C^{n}}$$

where QE_max_, *K*_d_, *C*, and *n* are the maximum QE, apparent *K*_d_, protein concentration, and Hill coefficient, respectively.^23,24^

Fluorescence spectra of varying concentrations of HisAb-Atto643 without and with G13-His(10%) are shown in Figure 3A, while those for all other G13-His conjugates are given in Figure S14 in the SI. Without G13-His(10%), the IF of HisAb-Atto643 increases linearly with the increasing *C*. In the presence of G13-His(10%), however, the relationship deviates increasingly from linearity, indicating that a growing fraction of the added HisAb-Atto643 is bound to G13-His and quenched (Figure 3B). G13-His(1–5%) conjugates gave the same trend of quenching but at lower levels than G13-His(10%). QE depended strongly on His_6_ density, with G13-His(10%) producing the highest QE and G13-His(1%) the lowest QE. Their different quenching behaviors are clearer from the corresponding QE–*C* plots (Figure 3C). For G13-His(10%), QE increases rapidly with increasing *C* and almost saturates at 1 nM, indicating very strong binding. In contrast, the QE–*C* response of G13-His(1%) is almost identical to that of G13-OH, the negative control without His_6_ antigens for HisAb binding and effectively resisting nonspecific interactions with proteins.^23,26,27^ This result suggests no measurable binding between G13-His(1%) and HisAbs under our experimental conditions (Figure 3C). The resulting QE–*C* plots and fittings by Hill equation for G13-His(2–10%) are shown in Figure 3C (*R* ^2^ > 0.99 for all fits). The fitting parameters are summarized in Table 2.

**Figure 3:**
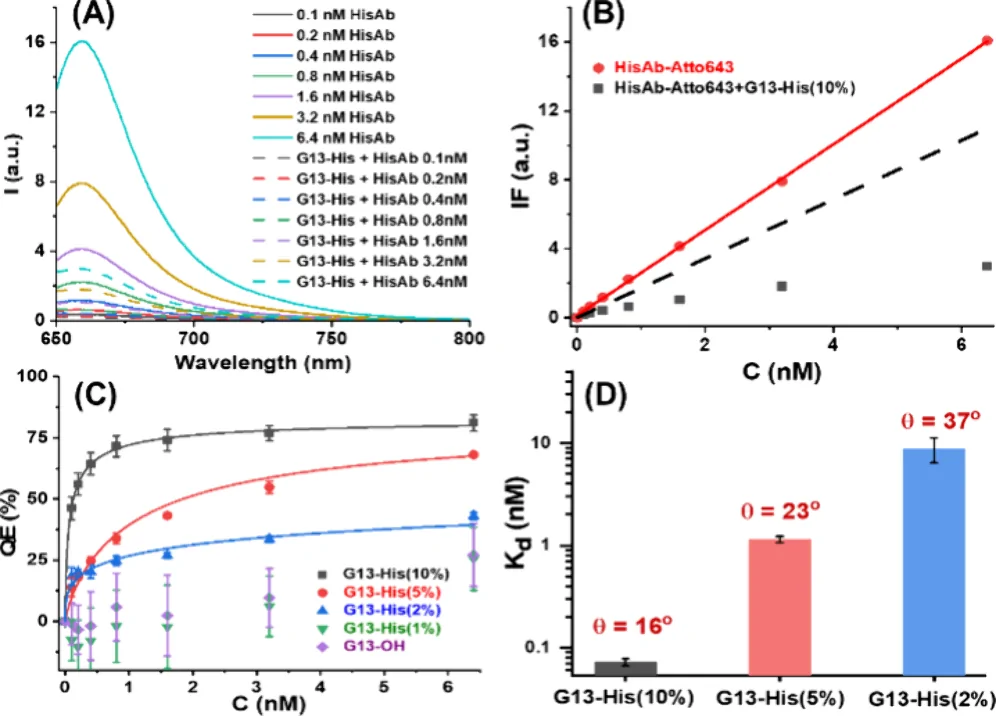
(A) Fluorescence spectra of HisAb-Atto643 without (solid lines) and with (broken lines) 1 mol equiv of G13-His(10%). The concentrations of both HisAb-Atto643 and G13-His(10%) were varied from 0.10 to 6.4 nM, but their molar ratio was fixed at 1:1. (B) IF vs *C* plots for HisAb-Atto643 without and with 1 mol equiv of G13-His(10%). (C) QE vs *C* plots for HisAb-Atto643 binding to 1 molar ratio of G13-His conjugates, fitted by Hill’s equation; where QE_max_ = 83.5%, obtained from fitting the G13-His(10%) data with a free float, was used to fit the G13-His(5%) and (2%) data (*R* ^2^ > 0.992 for all fits). G13-His(1%) gives the same QEs as the G13-OH negative control, hence its data were not fitted. Errors represent experimental errors. (D) Comparison of apparent *K*_d_ values for HisAb binding to G13-His­(2, 5 and 10%) of varying deflection angles (θ).

**Table 2:** Summary of Key Binding Parameters between G13-His­(0-10%) Conjugates and HisAb-Atto643 Obtained via the GNP Fluorescence Quenching Assay

His_6_%	θ (deg)	*d* (nm)	Apparent *K*_d_ (nM)	QE_max_	*n*
0	–	–	–	–	–
1	51.7 ± 2.5	8.4 ± 0.4	–	–	–
2	36.6 ± 1.7	6.3 ± 0.3	8.8 ± 2.5	83.5%	0.35 ± 0.05
5	23.1 ± 1.1	4.4 ± 0.2	1.14 ± 0.08	83.5%	0.84 ± 0.04
10	16.4 ± 0.8	3.3 ± 0.2	0.072 ± 0.006	83.5%	0.70 ± 0.08

HisAbs binding to G13-His conjugates is strong (sub- to low-nM apparent *K*_d_values) and highly dependent on His_6_ density (Table 2). Increasing the His_6_ density 5-fold (from 2% to 10%) reduces the apparent *K*_d_ from ∼8.8 to 0.072 nM, corresponding to >120-fold increase in affinity. Interestingly, the affinity of G13-His(2%) for HisAb is comparable to that of a single His-tagged protein binding to surface-immobilized anti-His antibodies measured by SPR (*K*_d_ ≈ 8.8 vs 9.4 nM).^36^ This result indicates that HisAb binding to G13-His(2%) is mainly monovalent, whereas binding to G13-His(10%) is dominated by chelative interactions on the same particle, resulting in much higher affinity. This result is fully consistent to that obtained from DLS in the previous section (Figure 2).

Interestingly, the DLS and fluorescence quenching assays gave quite different results for G13-His-HisAb binding. DLS shows that only G13-His(10%) gives significant *D*_h_ increases upon His-Ab binding, whereas the latter detects measurable HisAb-Atto643 quenching (above the G13-OH control) for all G13-His conjugates with ≥2% His_6_ density, with G13-His(10%) giving the strongest quenching. The discrepancy likely reflects the different readouts of the two methods: DLS may only detect stable, long-lived complexes formed by chelative binding, but not short-lived, dynamic structures formed by weak monovalent interactions. Because stable antibody–antigen complexes are required to trigger immune responses, the DLS-derived binding results are thus well-suited for assessing this capability. In contrast, the highly sensitive GNP fluorescence-quenching assay can detect both strong chelative and weak monovalent interactions and quantify their apparent *K*_d_ values, allowing us to capture the full range of binding strengths. By combining the binding and antigen presentation data, strong chelative Abs binding appears to only occurs at small θ values (≤16°) while weak to intermediate interactions are detectable at θ values up to 37°. The weaker interactions are likely to arise from mainly monovalent binding with occasional bivalent interactions with randomly positioned nearby epitopes. Given the *d* values in all G13-His(1–10%) are within the reported accessible range of Abs, and that strong binding is only detected at θ ≤ 16° with no detectable binding at θ ≥ 52°, these results indicate that θ is the dominant factor governing antibody binding to antigens displayed on solid nanoparticle surfaces.

## Conclusions

In summary, we varied the surface density of a His_6_ antigen on G13 scaffold to study how interantigen spacing (*d*), antigen density, and deflection angle (θ) influence antibody binding, using DLS and a GNP-based fluorescence-quenching assay. DLS detected binding only for G13-His(10%), whereas the GNP quenching assay detected and quantified binding for G13-His conjugates with 2–10% His_6_ density. We revealed a >120-fold increase in antibody affinity as His_6_ density increased from 2% to 10%, likely due to a transition from weak monovalent to strong chelative antibody binding. While the *d* values in all the G13-His(1–10%) conjugates fall within the reported accessible range of Abs’ two Fabs on flat surfaces, increasing *θ* for nanoparticle-displayed antigens signficantly weakened their antibody affinity. Specifically, strong chelative antibody binding occurs only at small θ (≤16°), whereas large θ (>37°) disfavors this binding mode, leading to only weak monovalent interactions. Our results reveal that *θ* is a dominant factor governing antibody binding to antigens displayed on solid nanoparticle surfaces. Overall, antibody binding requires higher antigen densities, reflected in smaller *d* and θ, on curved nanoparticles over that on flat surfaces.

## Supplementary Material



## Data Availability

All data associated with this paper are contained within the manuscript and electronic Supporting Information (SI). For the purpose of open access, the authors have applied a Creative Commons Attribution (CC BY) license to any Author Accepted Manuscript arising from this submission.

## Notes

Materials and Methods, synthesis and spectroscopic characterization of LA-EG_n_-based ligands, *D*_h_ histograms of G13-His conjugates of varying His_6_ densities before and after mixing with varying concentrations of HisAbs, fluorescence spectra of varying concentrations of HisAb in the absence and presence of 1 mol equiv of G13-His conjugates of varying His_6_ densities (PDF)
