# Supplementary material for: Antibody Binding to Peptide Conjugated Gold Nanoparticles Preferring Dense Antigen Coating with Small Deflection Angle
Source: ACS Nanosci Au. 2026 Jul 7;6(5):857–62. doi: 10.1021/acsnanoscienceau.6c00049 (PMC13693245; doi:10.1021/acsnanoscienceau.6c00049)
Supplement: Supplementary file 1 [file ng6c00049_si_001.pdf]

Supporting Information (SI)

Antibody Binding to Peptide Conjugated Gold Nanoparticles Preferring Dense Antigen Coating with Small Deflection Angle

Xinyu Ning,† Yuan Guo ‡,* and Dejian Zhou †,*

† School of Chemistry, Astbury Centre for Structural Molecular Biology and Bragg Centre for Materials Research, University of Leeds, Leeds LS2 9JT, UK. Email: d.zhou@leeds.ac.uk.

‡ School of Food Science and Nutrition and Astbury Centre for Structural Molecular Biology, University of Leeds, Leeds LS2 9JT, UK. Email: y.guo@leeds.ac.uk.

Materials and Methods

Materials

The tri-sodium citrate (99.5%) was purchased from Fisher Scientific, UK. The hydrogen tetrachloroaurate (iii) trihydrate (99.9%), tris[(1-benzyl-1H-1,2,3-triazol-4-yl)methyl]amine (TBTA) were purchased from Alfa Aesar, UK. The hydrochloric acid (~37%, Fisher Scientific) and nitric acid (68-70%, Sigma Aldrich) were used for preparing aqua regia in glassware cleaning prior to GNP synthesis. The $CuSO_4 \cdot 5H_2O$ (99.9%), sodium ascorbate (≥98%), phosphate buffered saline (PBS) tablet, maleimido-EG_4-C≡CH (90%) and N_3-EG_2-OH (>98%) were purchased from Sigma Aldrich, UK. Bovine serum albumin (BSA, 99%) was purchased from BioServ, UK. His_6-Cys peptide (>98%, HPLC purified) was purchased from China peptides. Antibody against His-tag were purchased from R&D Systems, Bio-Techne, UK. Deionised water was obtained from the ELGA Purelab classic UVF system (>18.2 mΩ cm^{-1}) and used in all experiments.

Synthesis of LA-EG_{11}-EG_4-His_6 and LA-EG_6-EG_2-OH ligands

LA-EG_{11}-EG_4-His_6 was synthesised in two steps (**Scheme S**1): first, to obtain the linker LA-EG_{11}-EG_4-Mal, 15 mg (0.048 mmol) maleimido-EG_4-C≡CH was added to a 5 mL round bottom flask. For every 1 molar equivalent maleimido-EG_4-C≡CH, 40 mg LA-EG_{11}-N_3 (1.1 molar equivalents, synthesised by a former member, Dr Darshita Budhadev) were added to the flask. Then mixed solvent of tetrahydrofuran (THF) / water (1:1 v/v) were added to dissolve the above reagents with stirring. After reagents were fully dissolved, 0.4 mg $CuSO_4 \cdot 5H_2O$ (0.036 molar equivalents), 1.6 mg tris[(1-benzyl-1H-1,2,3-triazol-4-yl)methyl]amine (TBTA, 0.063 molar equivalents), and 1.3 mg sodium ascorbate (0.135 molar equivalents) were added as catalysts with stirring. The reaction was performed at room temperature overnight. Then the product was purified using a P2 Bio-Gel size exclusion column to obtain 44.4 mg of the desired LA-EG_{11}-EG_4-Mal ligand in a yield of approximately 86%. Second, 22.2 mg of LA-EG_{11}-EG_4-Mal (0.021 mmol) was weighted and dissolved in mixed solvent of EtOH/H_2O (50%/50%), and then 24 mg of His_6-Cys peptide (1.2 molar equivalent to maleimide) was added. The mixture was shaken and allowed to react at room temperature overnight. After the reaction, the mixture was purified using a P2 Bio-Gel size-exclusion column to yield 10.4 mg of the desired LA-EG_{11}-EG_4-His_6 ligand with a yield of approximately 25%.

LA-EG_6-EG_2-OH was synthesised by copper catalysed azide-alkyne click reaction as reported previously (**Scheme S**1 B).[1] Briefly, 30 mg LA-EG_6-C≡CH (provided by a former member, Dr Darshita Budhadev) was weighted in a 5 mL round bottom flask. For every 1 molar equivalent LA-EG_6-C≡CH, 1.1 molar equivalents of N_3-EG_2-OH were added to the flask, respectively. Then mixed solvent of THF / water (1:1 v/v) was added to dissolve the above reagents with stirring. After reagents were fully dissolved, 0.036 mol equivalents $CuSO_4 \cdot 5H_2O$, 0.063 mol equivalents TBTA, and 0.135 mol equivalents sodium ascorbic acid were added as catalysts with stirring. Reaction performed at room temperature for overnight. Then the product was

purified using a P2 Bio-Gel column to obtain the desired LA-EG_6-EG_2-OH ligand at a yield of approximately 36%.

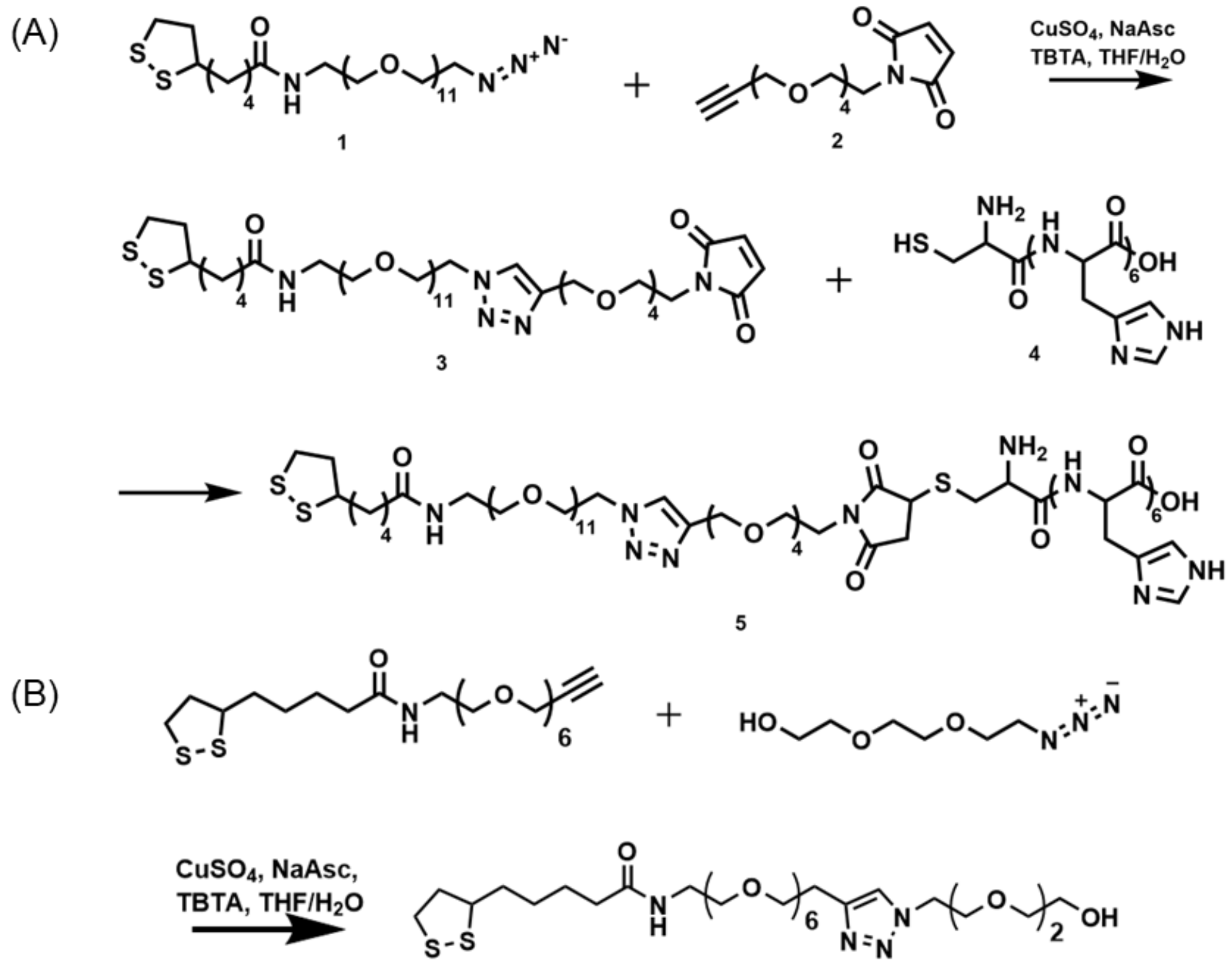

Scheme S1. The synthetic routes to (A) LA-EG_{11}-His_6 and (B) LA-EG_6-OH. (**A**) LA-EG_{11}-Mal (full form LA-EG_{11}-EG_4-Maleimide, compound 3) was obtained *via* copper-catalysed click chemistry using LA-EG_{11}-N_3 (compound 1) and maleimido-EG_4-C≡CH (compound 2) with $CuSO_4 \cdot 5H_2O$, TBTA, NaAsc as the catalysts and THF/H_2O (1:1 v/v) as the solvent, yielding ~86%; then LA-EG_{11}-His_6 (full form LA-EG_{11}-EG_4-His_6, compound 5) was obtained *via* maleimide-thiol reaction using LA-EG_{11}-Mal (compound 3) and Cys-His_6 (compound 4), yielding ~25%. (**B**) LA-EG_6-OH (full form LA-EG_6-EG_2-OH) was obtained *via* copper-catalysed click chemistry using LA-EG_6-C≡CH and N_3-EG_2-OH with $CuSO_4 \cdot 5H_2O$, TBTA, NaAsc as the catalysts and THF/H_2O (1:1 v/v) as the solvent, yielding ~36%.

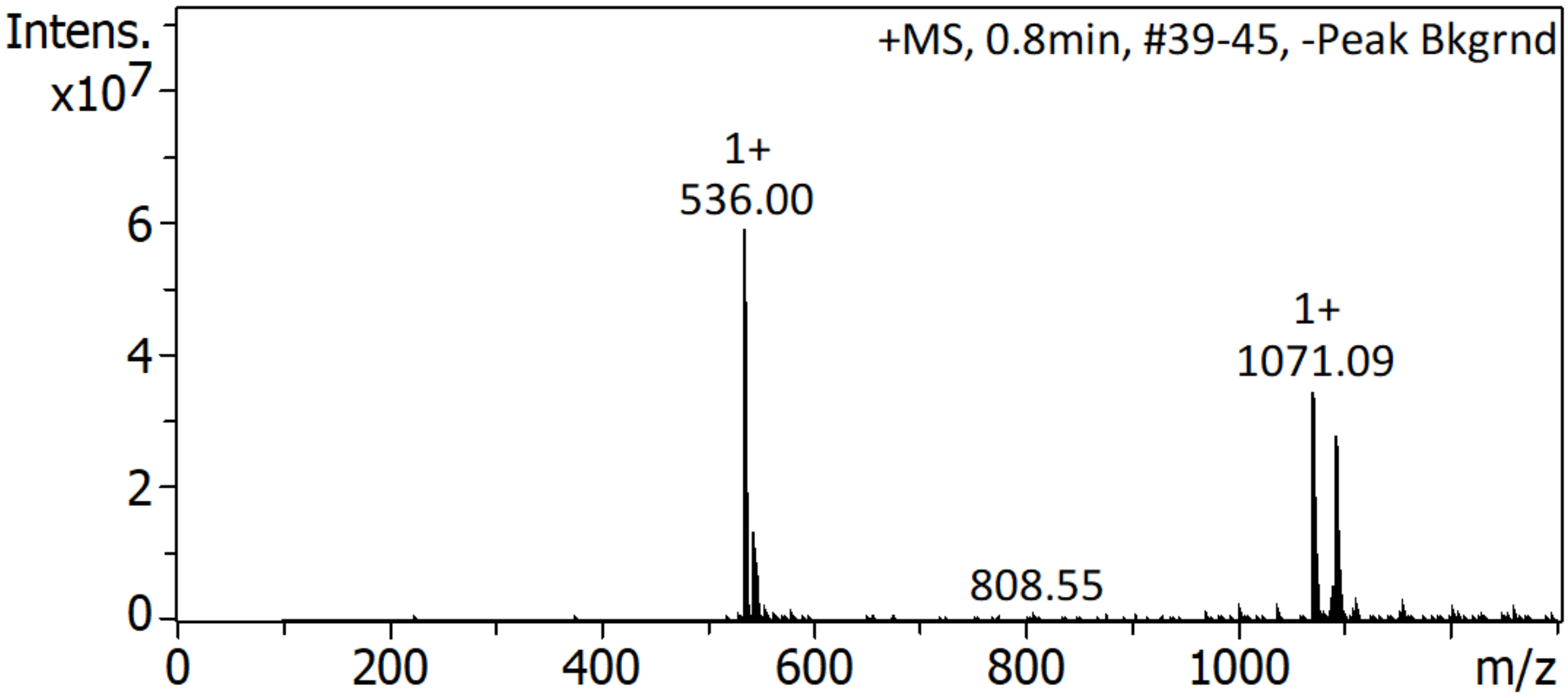

Figure S1. LC-MS spectrum of LA-EG$_{11}$-EG$_4$-Maleimide. Calculated *m/z* for $C_{47}H_{83}N_5O_{18}S_2$ $[M+H]^+$ 1070.53; found 1071.09. The peak at 536.00 corresponds to $[M+2H]^{2+}$.

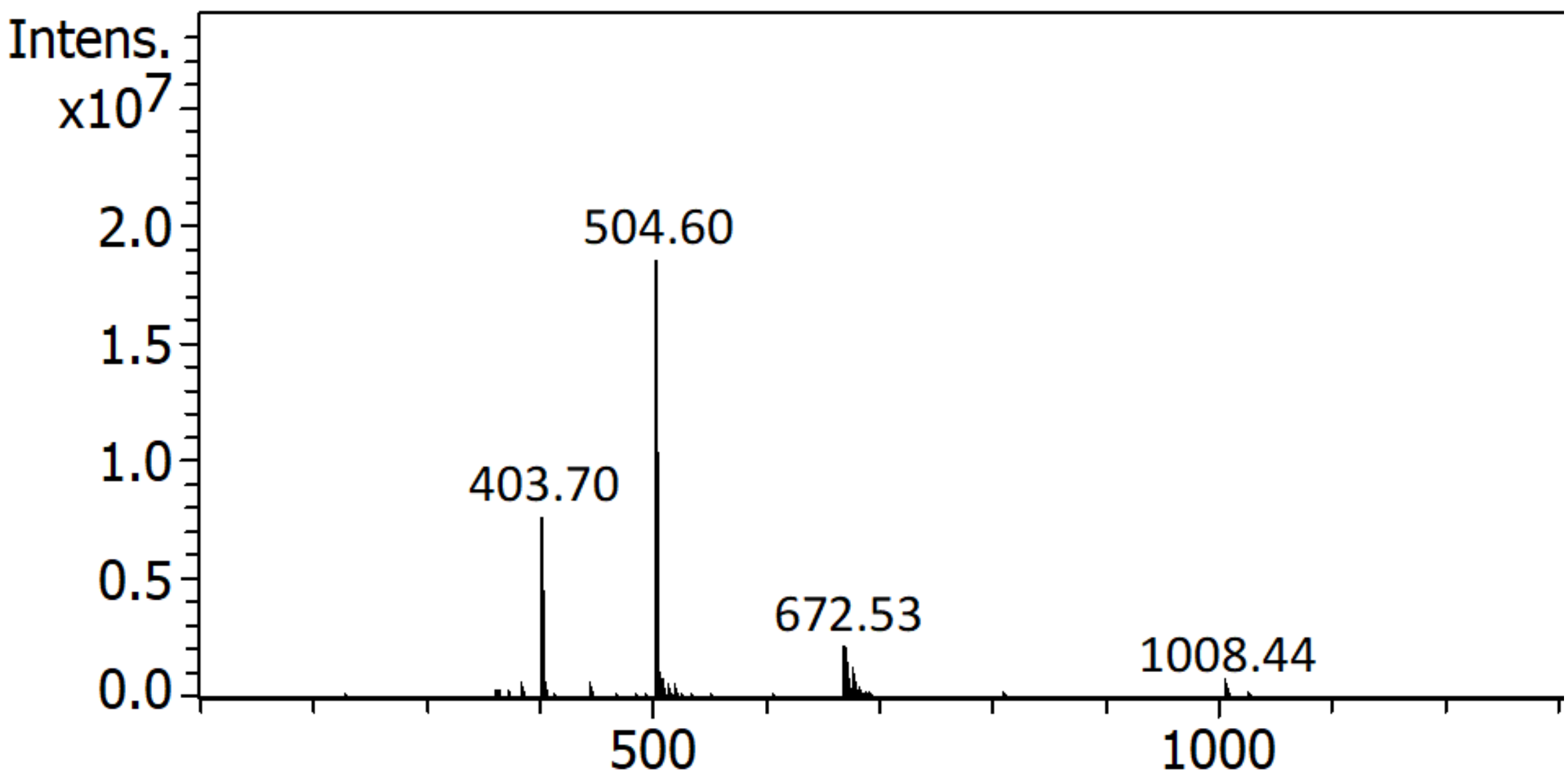

Figure S2. LC-MS spectrum of LA-EG_{11}-EG_4-His_6. Calculated *m/z* for $C_{86}H_{132}N_{24}O_{26}S_3$ $[M+2H]^{2+}$ 1008.45; found 1008.44. The peaks at 672.53, 504.60 and 403.70 correspond to $[M+3H]^{3+}$, $[M + 4H]^{4+}$ and $[M + 5H]^{5+}$, respectively

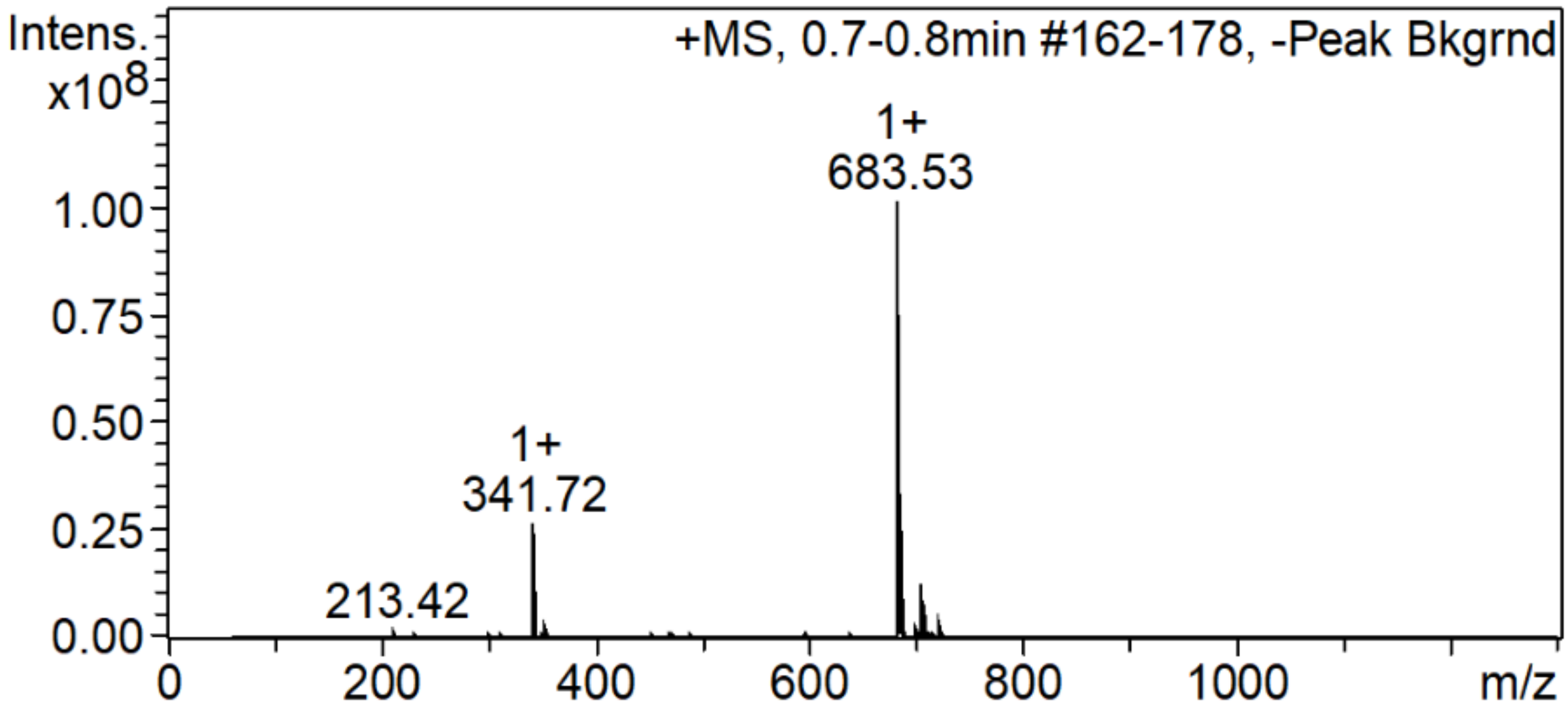

Figure S3. LC-Mass spectrum of LA-EG$_6$-EG$_2$-OH. LCMS: calculated *m/z* for $C_{29}H_{54}N_4O_{10}S_2$ $[M+H]^+$ 683.33; found 683.53.

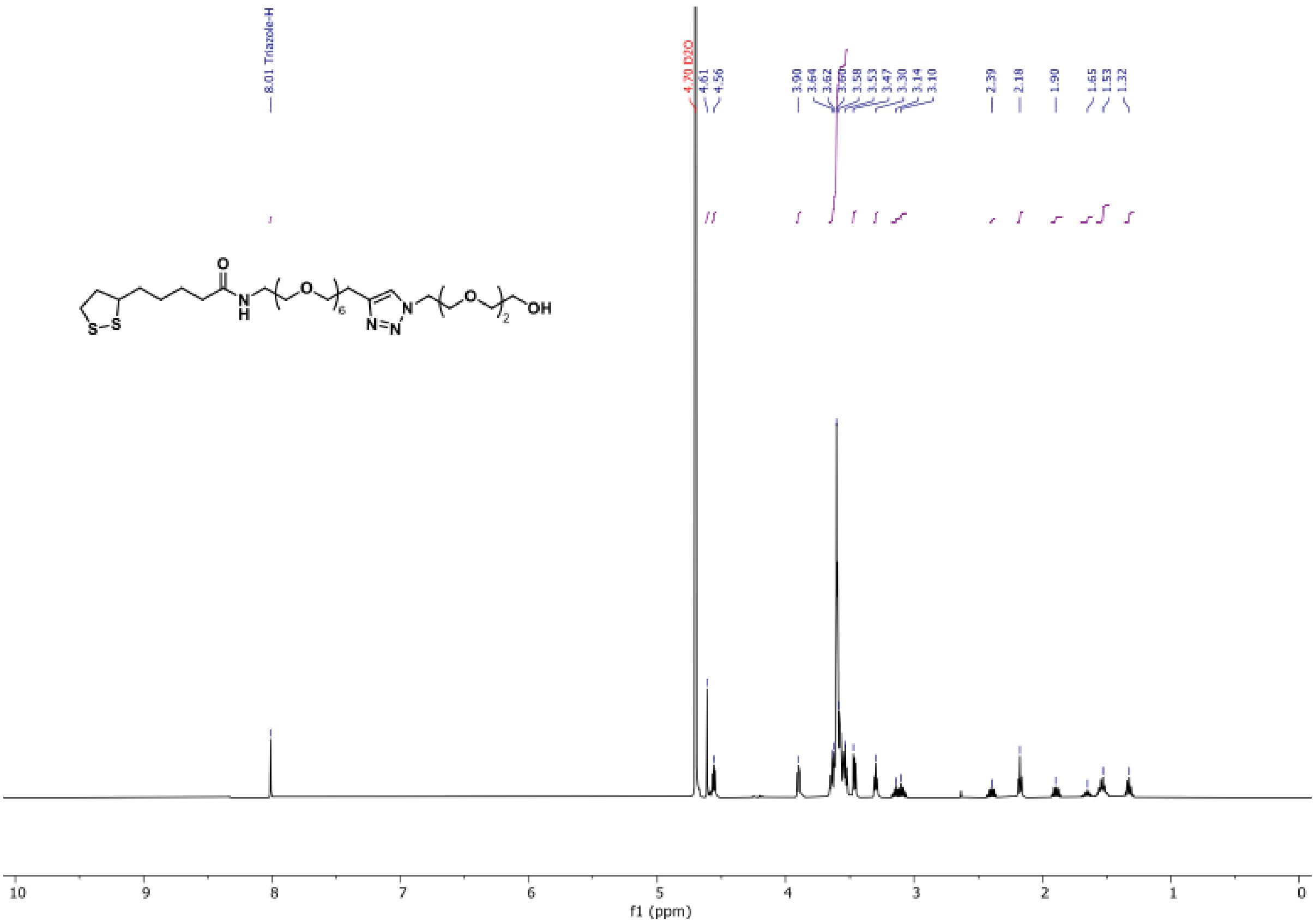

Figure S4. ^{1}H NMR (500 MHz) spectrum of LA-EG$_6$-EG$_2$-OH in D_2O. δ (ppm): 8.01 (s, 1H), 4.61 (s, 2H), 4.56 (t, 2H), 3.90 (t, 2H), 3.66 – 3.52 (m, 31H), 3.48 – 3.45 (m, 2H), 3.30 (t, 2H), 3.17 – 3.06 (m, 2H), 2.39 (s, 1H), 2.18 (t, 2H), 1.93 – 1.85 (m, 1H), 1.70 – 1.61 (m, 1H), 1.58 – 1.48 (m, 3H), 1.36 – 1.29 (m, 2H).

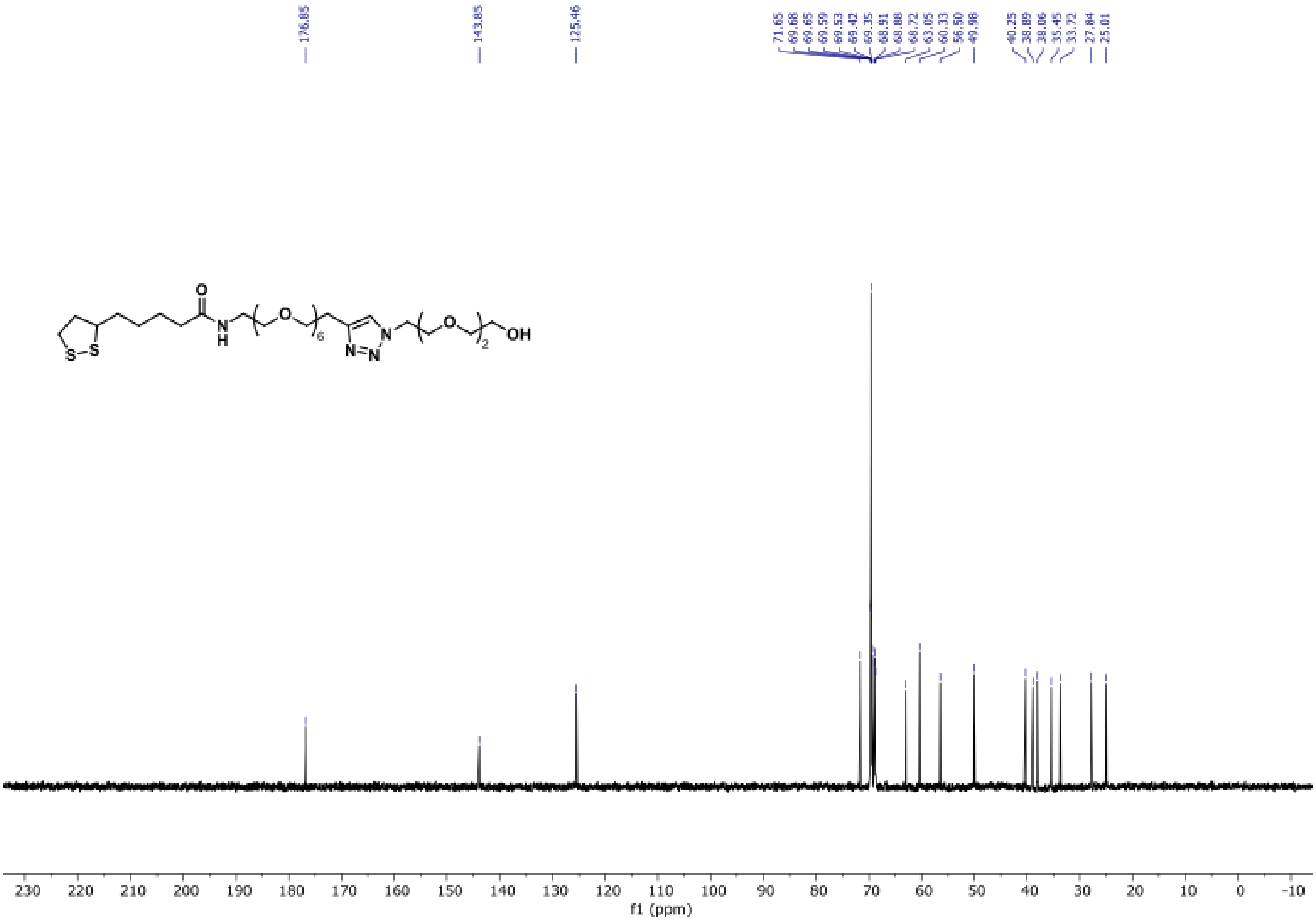

Figure S5. ^{13}C NMR spectrum (125 MHz) of LA-EG$_6$-EG$_2$-OH in D_2O. δ (ppm) 176.85 (C=O), 143.85, 125.46, 71.65, 69.68, 69.65, 69.59, 69.53, 69.42, 69.35, 68.91, 68.88, 68.72, 63.05, 60.33, 56.50, 49.98, 40.25, 38.89, 38.06, 35.45, 33.72, 27.84, 25.01.

Synthesis of 13 nm gold nanoparticles

Spherical gold nanoparticles with an average diameter of ~13 nm (G13s) were synthesised by the standard citrate reduction method as reported previously.[2] First, 79 mg of chloroauric acid trihydrate ($HAuCl_4 \cdot 3H_2O$) was weighed into a washed glass vial, transferred to a cleaned 250 mL three-neck round-bottom flask fitted with a stir bar, and dissolved in 200 mL of de-ionised water. The flask was placed in an oil bath under vigorous stirring (> 600 rpm) and heated to reflux with an air condenser. Separately, 228 mg of tri-sodium citrate was dissolved in 10 mL of water. When the aqueous solution started to boil, the citrate solution was quickly injected; the colour of the mixture turned from yellow to wine red within 1–2 min. Heating was continued for a further 20 min, after which the heat source was removed while stirring was maintained in the bath for an additional 40 min. Finally, the flask was taken out of the oil bath and allowed to cool to room temperature. This produced citrate stabilized spherical gold nanoparticles of ~13 nm in diameter as confirmed by TEM (see below).

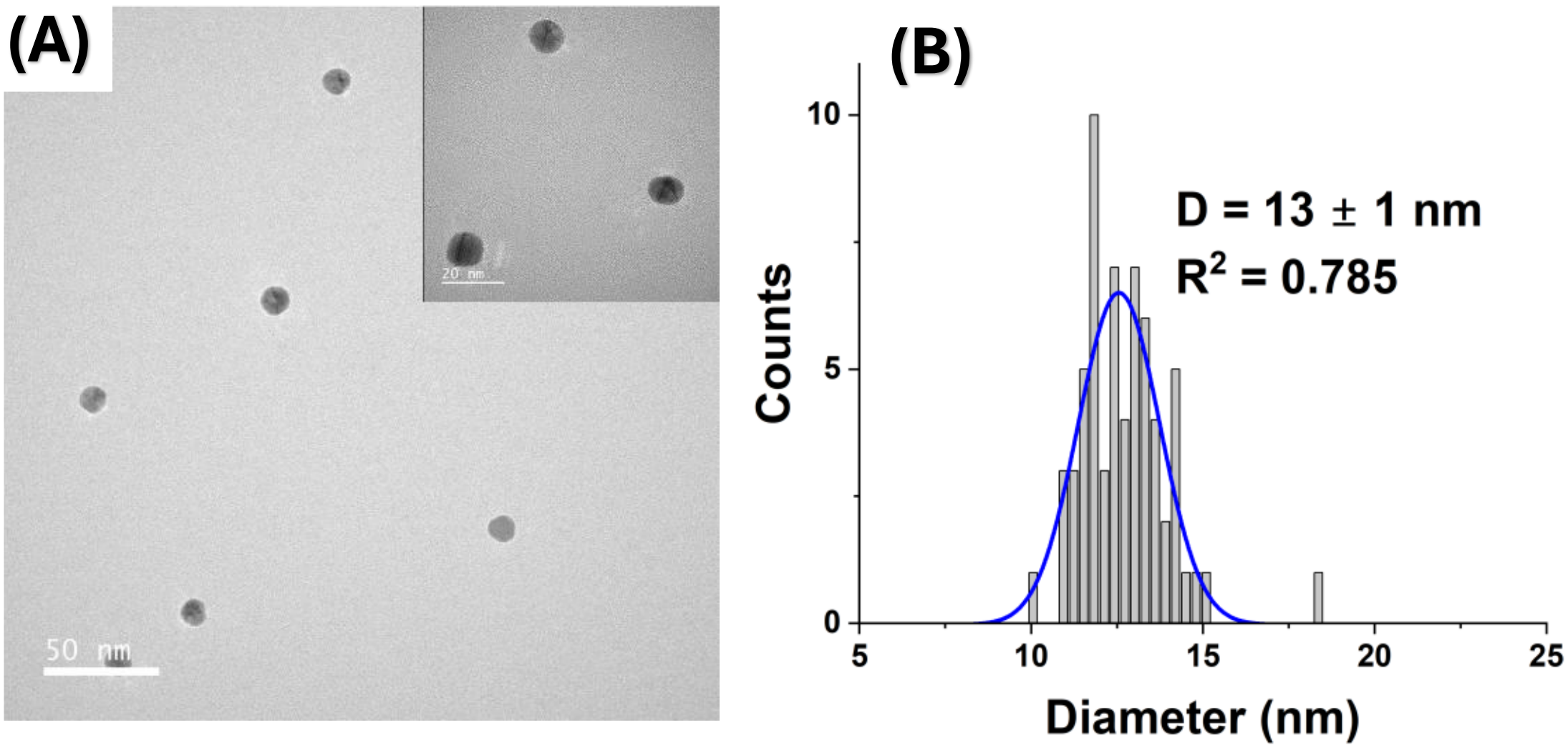

Figure S6. (**A**) A typical TEM image of citrate stabilised G13 (G13-citrate) and (**B**) statistical analysis of the GNP core size, giving an average diameter of 13 ± 1 nm (mean ± ½ FWHM). The same batch of G13-citrate was used here to make G13-His_6 conjugates as that of the G13-glycan conjugates reported previously (see Ning, X., et al. *JACS Au*, 2024, **4**, 3295).

Estimation of LA-ligand: G13 molar ratio (LGMR) required to fully coat G13

Total gold surface area of one G13 particle,

$A_{G13} = 4\ \pi\ r^2 = 4 \times 3.14 \times (13\ nm/2)^2 = 530\ nm^2$

Assuming that the LA-ligand self-assembly on G13 surface is the same as that of alkanethiol self-assembly on a flat Au(111) surface, with each thiol group occupying a 3-fold hollow site, (see Ulman, A. *Chem. Rev.* **1996**, *96*, 1533) then each thiol group would occupy a surface area,

$A_{Th} = 0.214\ nm^2$

Each LA based dithiol ligand forms two Au-S bonds upon binding to G13, thus it should occupy twice the surface area of an alkanethiol on gold surface,

$A_{LA} = 2\ A_{Th} = 2 \times 0.214\ nm^2 = 0.428\ nm^2$

The number of LA-based ligand (M) to fully coat a G13 particle by forming a self-assembled monolayer:

$M = A_{G13}/A_{TA} = 530/0.428 = \sim 1240$

This calculation indicates that a total LA-ligand: G13 molar ratio (LGMR) of 3000:1 would give ~2.4 times (e.g., 3000/1240 = ~2.4) the amounts of ligands required to coat the G13 surface by forming a complete self-assembled monolayer.

Preparation of G13-His_6 conjugates

All G13-His_6 conjugates were prepared under a total ligand (LA-EG_{11}-EG_4-His_6 + LA-EG_6-EG_2-OH) : G13 molar ratio (LGMR) of 3000 : 1. By systematically varying the LA-EG_{11}-EG_4-

His_6 ligand content in the ligand mixture from 15%, 10%, 5%, 2%, 1%, to 0% (with the rest being the LA-EG_6-EG_2-OH control spacer ligand), the G13 surface LA-EG_{11}-EG_4-His_6 density was varied from 15% (with 15% of the total ligands being LA-EG_{11}-EG_4-His_6) to 0% (with 100% LA-EG_6-EG_2-OH control ligand). For the conjugation procedure, half of the total ligand molar ratio (1500 molar equivalents) of LA-EG_6-EG_2-OH ligand was added to 1 molar equivalent of citrate stabilized G13 in a glass vial with sonication for 2 min. The reaction was kept undisturbed at room temperature for 6 h to partially passivate G13 surface to provide good enough stability for further peptide conjugation. Then the remaining ligand mixture was added with sonication for 2 min, and the conjugation solution was incubated at room temperature for a further 24 h to make G13-His_6 conjugates *via* self-assembly. After incubation, the resulting G13-His_6 conjugate solutions were transferred to Eppendorf tubes pre-treated with 0.2 % Tween 20 (v/v) aqueous solution, and then centrifuged at 17000 × g for 30 min. After careful removal of the clear supernatants, the resulting GNP residues were washed 3 times with deionised water, each including 30 min centrifugation at 17000 × g and followed by removal of clear supernatants. The final G13-His residues were dispersed in deionised water and their concentrations were determined from their UV-vis absorption at 522 nm (A_{522}, the maximum SPR absorption peak of G13) using the Beer-Lamber law using G13's absorption extinction coefficient of 2.32×10^8 $M^{-1}cm^{-1}$ (see Figure S7 below). Their hydrodynamic diameters were acquired *via* dynamic light scattering (DLS) on a Malvern Zetasizer Nano ZSP in disposable plastic cuvettes at 25 °C. The volume distribution histograms obtained from the instrumental software were fitted by the standard single Gaussian distribution function using OriginPro 2025.

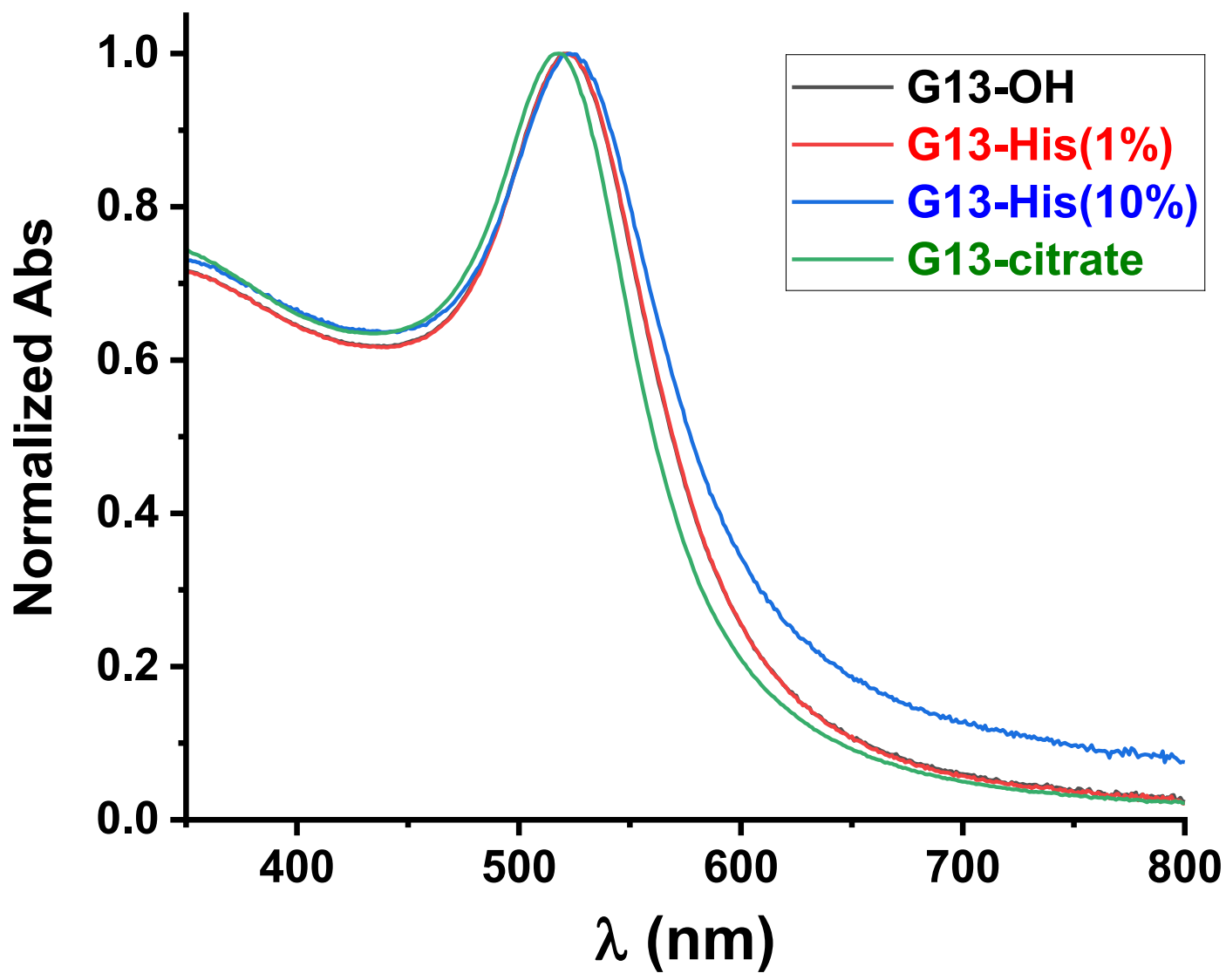

Figure S7: Normalized UV-vis absorption spectra of the citrate stabilized G13 (G13-citrate) and representative G13-His conjugates with His_6 densities of 0 (G13-OH), 1 and 10%. The maximum SPR absorption peaks for all UV-vis spectra are normalized to 1. G13-citrate gives a maximum SPR absorption peak at 518 nm, while those for all G13-His conjugates are the same, at ~522 nm, showing a redshift of ~ 4 nm.

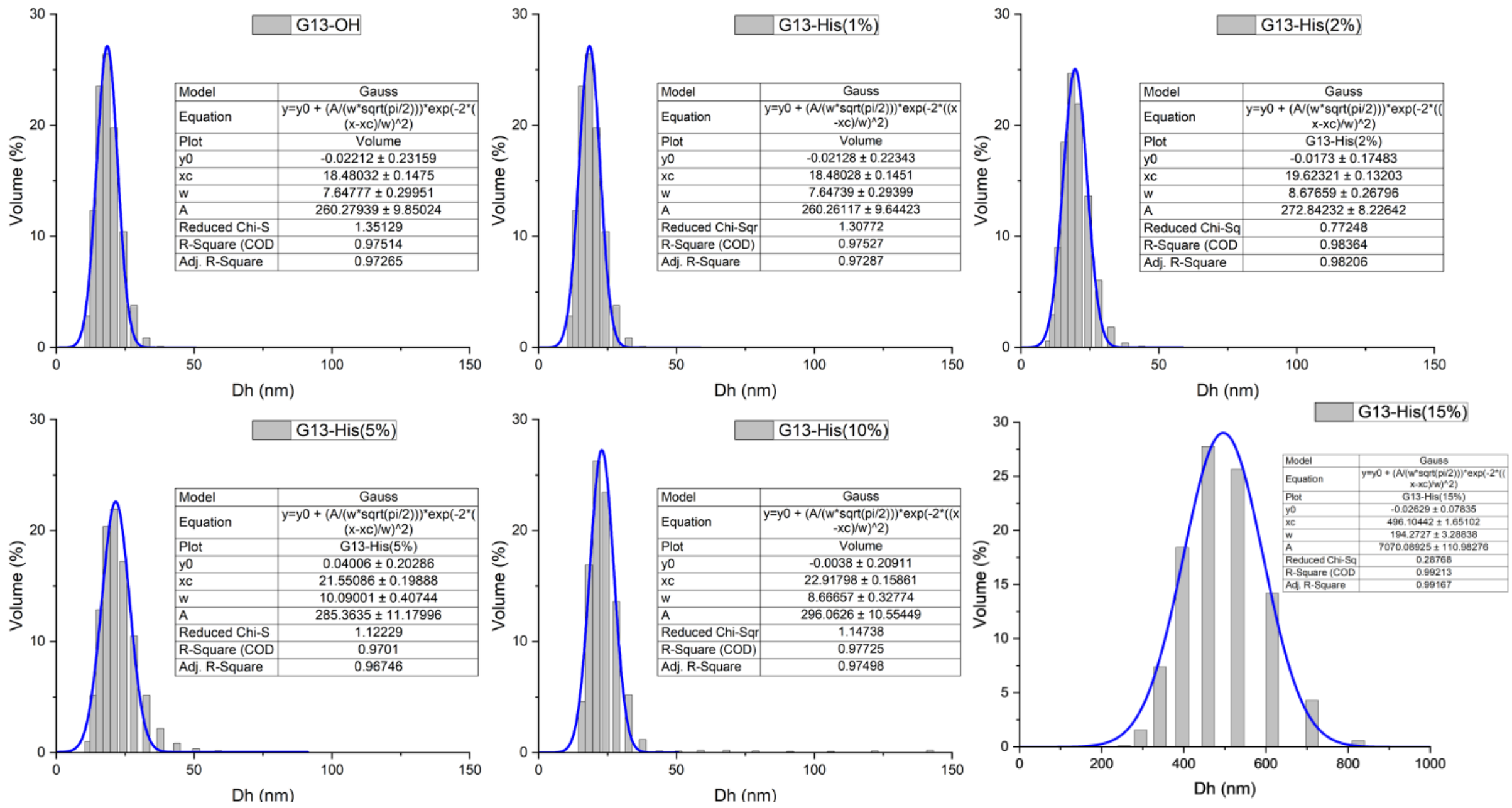

Figure S8. D_h (volume) distribution histograms fitted by Gaussian distribution for G13-His conjugates with varying His_6 densities, 0% (G13-OH), 1%, 2%, 5%, 10 to 15%, in a PBS buffer (137 mM NaCl, 2.7 mM KCl, 10 mM Na_2HPO_4, 1.8 mM KH_2PO_4, pH 7.4). The D_h of G13-His(15%) is much larger than all others (~406 nm *vs.* ~18 - 23 nm), a clear indication of aggregation.

Table S1: The D_h values (given as means ± ½ W, where W is the full-width at half maximum of Gaussian fit, see SI, Figure S8) of G13-His conjugates (0-10%) measured by DLS. *G13-His(15%) is unstable and readily aggregate in PBS, making it unsuitable for binding studies.

**G13-His conjugates**	**D_h (mean ± ½ W, nm)**	**R^2**
G13-OH	18.5 ± 3.8	0.973
G13-His(1%)	18.5 ± 3.8	0.973
G13-His(2%)	19.6 ± 4.4	0.982
G13-His(5%)	21.6 ± 5.1	0.967
G13-His(10%)	22.9 ± 4.4	0.975

Calculation of antigen presentation parameters

The surface area occupied by each LA-EG_{11}-EG_4-His_6 ligand (footprint k, in nm^2), the angle between two nearest His_6 epitopes (average deflection angle θ, in degrees), and the average inter-His_6 epitope distance (d, in nm) on G13 were estimated from the mean D_h values (peak width ± 1/2 W not considered) and His_6-ligand valencies (N, uncertainty considered) of the G13-His_6 conjugates using the method first reported by the Mirkin group.[3] The His_6-ligand valency (N, the number of His_6 ligand loaded on each G13) was estimated by assuming that

the LA-EG$_n$-His$_6$ ligand content on G13 surface is the same as that of the His$_6$ ligand content% in the ligand mixture used G13 conjugation, and the total number of LA-EG$_n$-based ligand on each G13 is the same as that of G13-LA-EG$_4$-psDiMan conjugate prepared under the identical LGMR of 3000 using the same G13 batch reported previously (1963 ± 186)[4]. Calculation was based on that all LA-EG$_n$-based ligands were distributed randomly on the G13 surface without phase separation using the following equations S1-S3: [3, 4]

$$k = \frac{\pi D_h^2}{N} \tag{S1}$$

$$\theta = \frac{360\sqrt{\frac{k}{\pi}}}{\frac{D_h}{2}\pi} = 229.3\sqrt{\frac{1}{N}} \tag{S2}$$

$$d = D_h \sin\left(\frac{\theta}{2}\right) \tag{S3}$$

where D_h is the hydrodynamic diameter of each G13-His$_6$ conjugate, and N is the average number of LA-EG$_{11}$-EG$_4$-His$_6$ ligand on each G13 surface.

DLS studies of G13-His$_6$ interaction with anti-His antibody

Prior to the binding study, wild-type antibody against His-tag (HisAb) was prepared in phosphate-buffered saline (PBS) buffer at a concentration of 4 nM, and its D_h was confirmed *via* DLS. To determine the interactions between G13-His$_6$ conjugates and HisAb *via* DLS, 2 nM G13-His$_6$ (with varying His$_6$ densities) was mixed with varying concentrations of HisAb in PBS to obtain the HisAb: GNP molar ratio of 1, 2, 4, 8, 16, and 32, respectively. All samples were prepared in disposable plastic cuvettes with to a final volume of 70 µL, incubated at room temperature for 30 min. Their hydrodynamic diameters (D_hs) were then recorded on a Malvern Zetasizer Nano ZSP in disposable cuvettes at 25 °C, and the volume distribution histograms obtained from the instrument software were fitted with standard Gaussian function using OriginPro 2025.

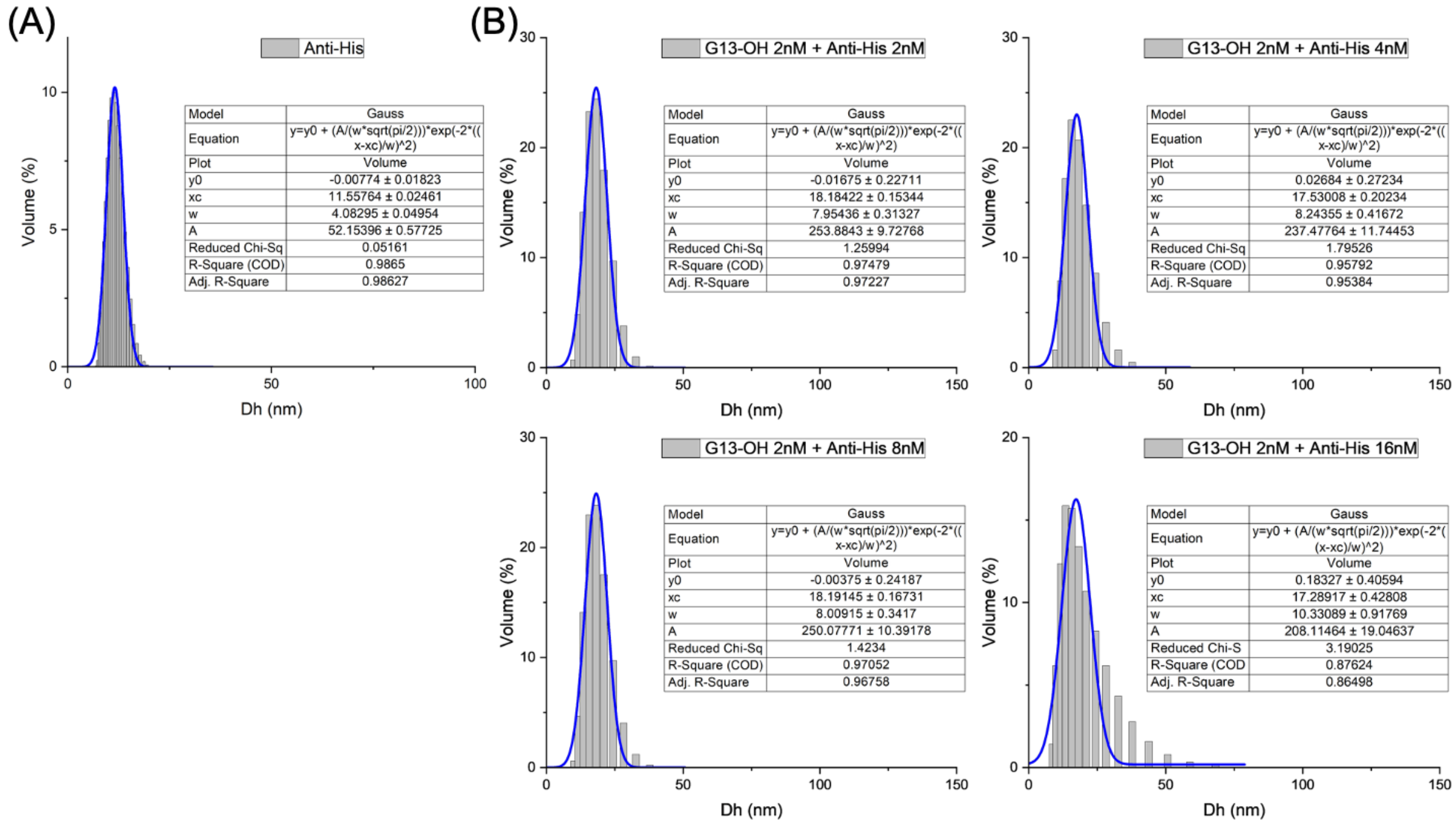

Figure S9. D_h (volume population) distribution histograms fitted by a single Gaussian distribution function for (**A**) HisAb alone, and (**B**) G13-OH (2 nM) after mixing with varying concentrations of HisAb (ranging from 2 to 16 nM) in PBS buffer.

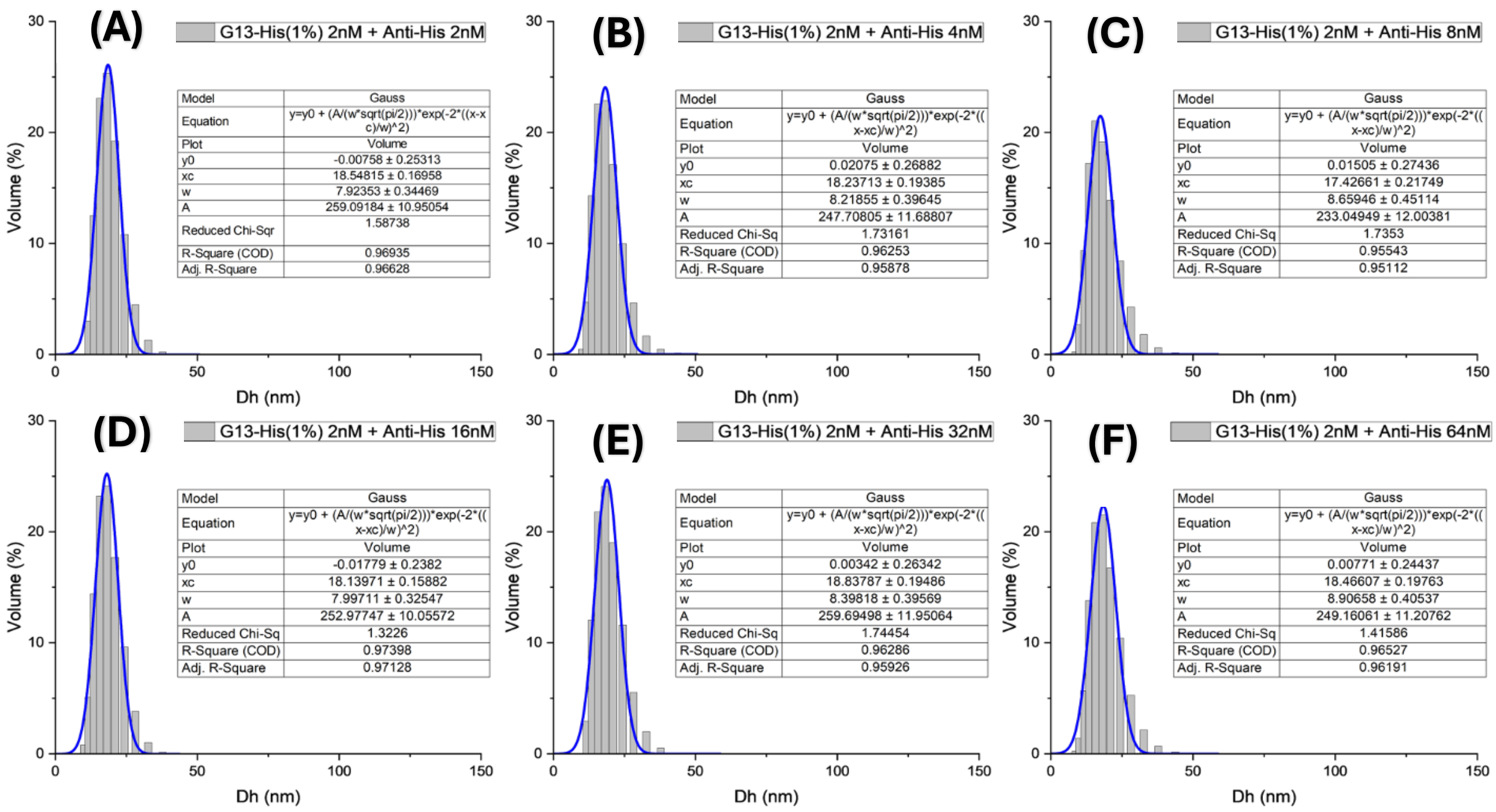

Figure S10. D_h (volume population) distribution histograms fitted by a single Gaussian distribution function for 2 nM G13-His(1%) after mixing with varying concentrations of HisAb in PBS buffer. (**A**) 2 nM; (**B**) (4 nM); (**C**) 8 nM; (**D**)16 nM; (**E**) 32 nM; (**F**) 64 nM.

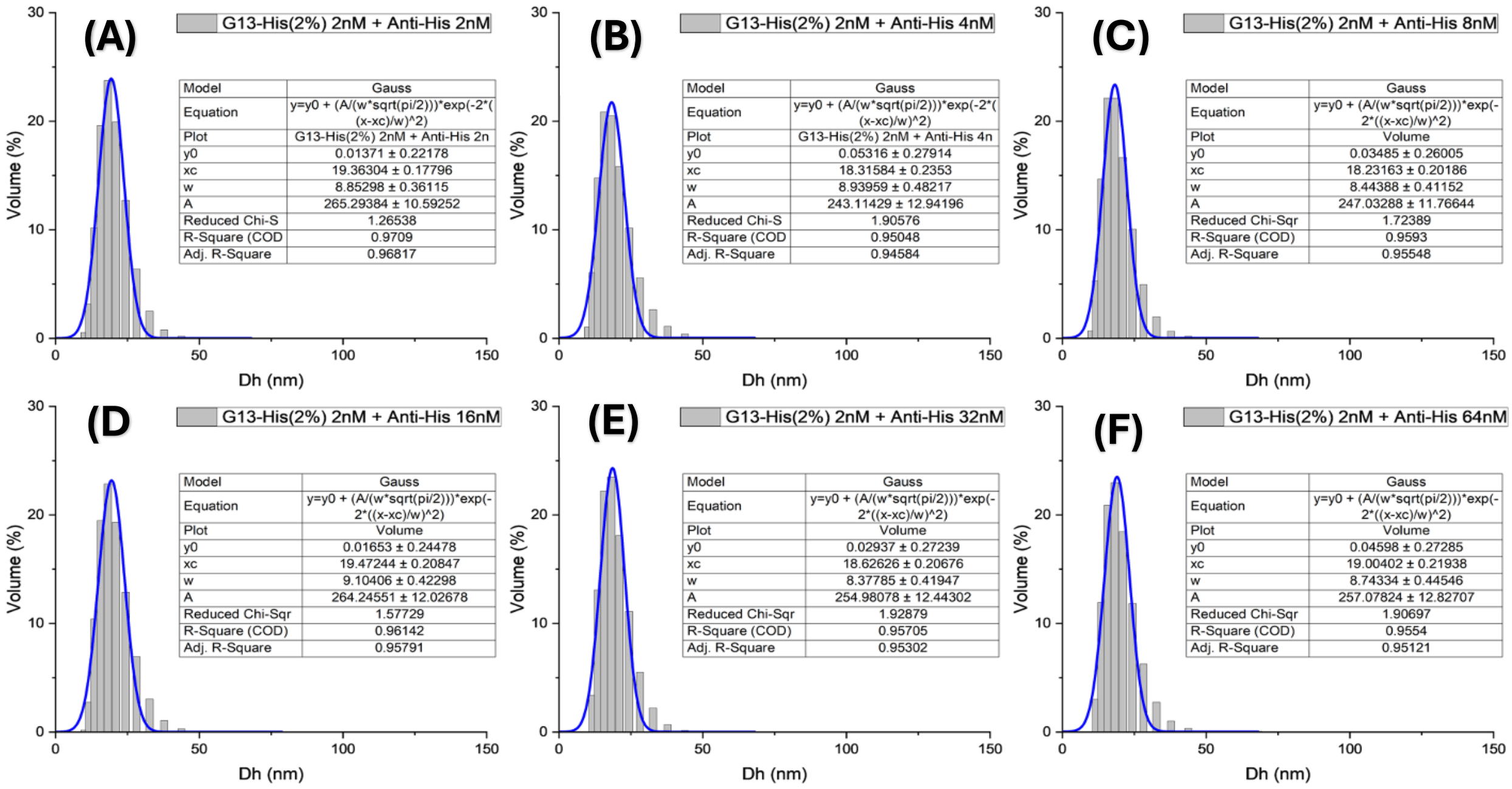

Figure S11. D_h (volume population) distribution histograms fitted by a single Gaussian distribution function for 2 nM G13-His(2%) after mixing with varying concentrations of HisAb in PBS buffer. (**A**) 2 nM; (**B**) (4 nM); (**C**) 8 nM; (**D**)16 nM; (**E**) 32 nM; and (**F**) 64 nM.

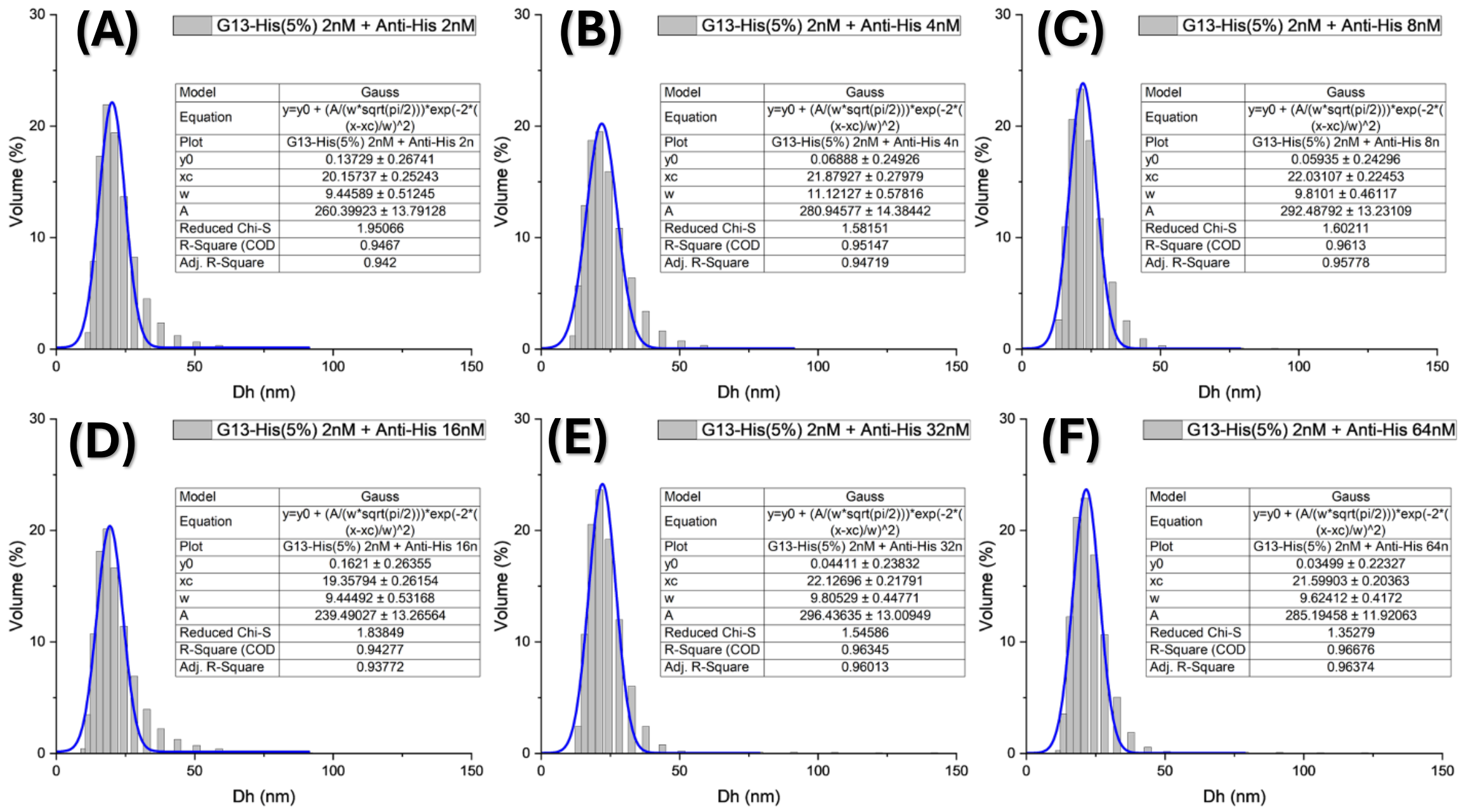

Figure S12. D_h (volume population) distribution histograms fitted by a single Gaussian distribution function for 2 nM G13-His(5%) after mixing with varying concentrations of HisAb in PBS buffer. (**A**) 2 nM; (**B**) (4 nM); (**C**) 8 nM; (**D**)16 nM; (**E**) 32 nM; and (**F**) 64 nM.

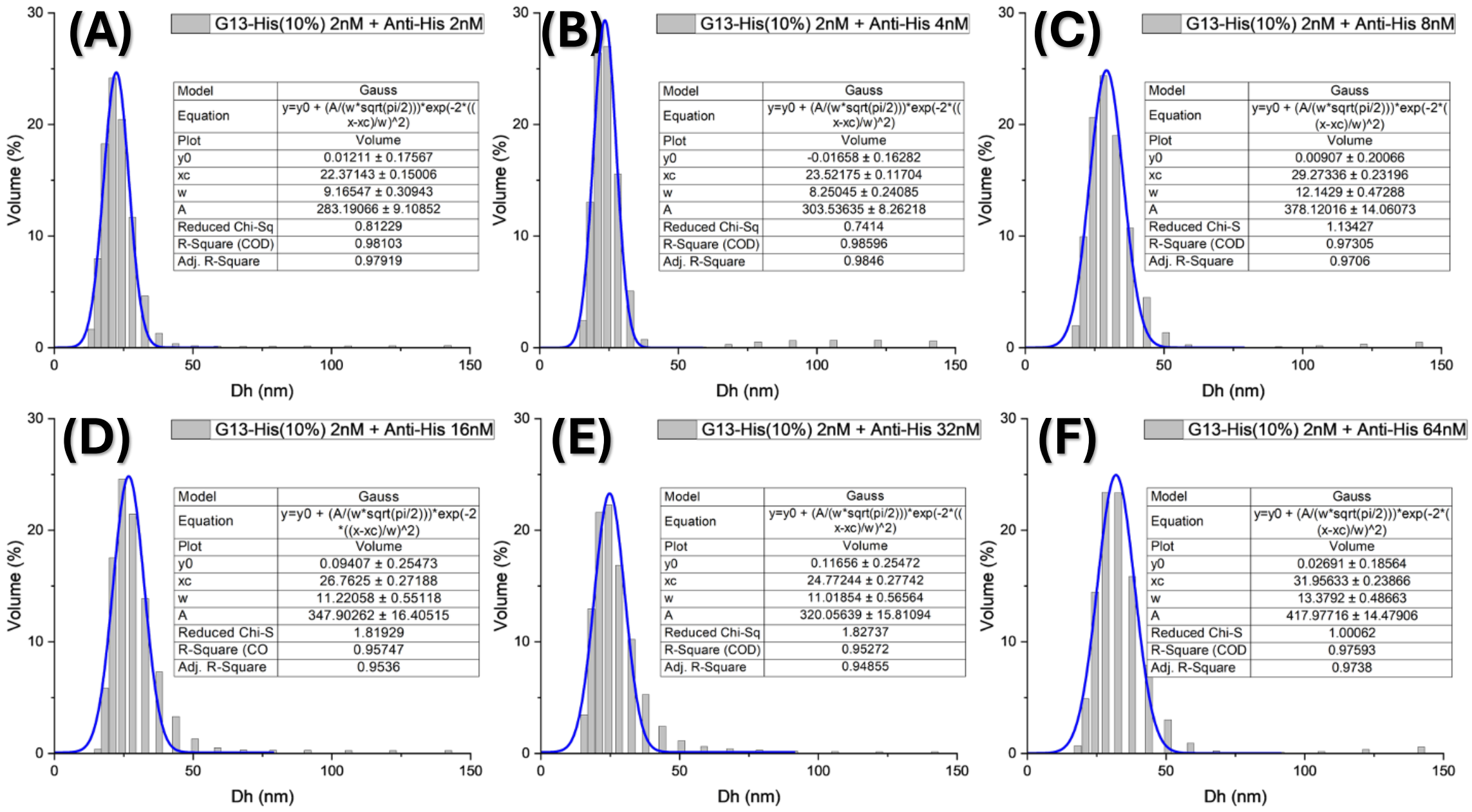

Figure S13. D_h (volume population) distribution histograms fitted by a single Gaussian distribution function for 2 nM G13-His(5%) after mixing with varying concentrations of HisAb in PBS buffer. (**A**) 2 nM; (**B**) (4 nM); (**C**) 8 nM; (**D**)16 nM; (**E**) 32 nM; and (**F**) 64 nM.

Antibody labeling

To obtain Atto643 dye labelled antibody against His-tag (HisAb-Atto643), HisAb was prepared to 2 mg/mL in a buffer containing 20 mM HEPES and 150 mM NaCl, pH 8.3. To 1 molar equivalent of HisAb (MW_{IgG} = 150 kDa), 5 molar equivalents of NHS ester activated Atto643 in dry DMSO was added. The mixture was vortexed immediately to mix, then the mixture was incubated at room temperature for 2 h. The reaction mixture was kept in a fridge at 4 °C overnight, then the mixture was washed using 3 kDa MWCO with PBS to remove any unlabelled free dyes. The concentration of labelled antibody was determined by a UV-vis spectrometer and calculated using the following equation:

$$Conc\ of\ IgG\ (mol/L) = \frac{A_{280} - A_{643} \times 0.04}{\varepsilon(IgG) \times L\ (cm)}$$

where A_{280} and A_{643} are the absorbance of the labeled antibody at 280 nm and the Atto643 dye at 643 nm from the UV-vis spectra, respectively. ε(IgG) is the extinction coefficient of IgG at 280 nm, 210,000 $M^{-1}cm^{-1}$; and the path length L = 1 cm. The contribution of Atto643 dye at A_{280} (A_{643} x 0.04) is corrected from the calculation. Moreover, the dye concentration in the final product can be determined using the following equation:

$$Atto643\ Conc\ (mol/L) = \frac{A_{643}}{\varepsilon(Atto643) \times L(cm)}$$

where A_{643} is the absorbance of the Atto643 dye at 643 nm (IgG does not absorb at this wavelength). The extinction coefficient $\varepsilon_{Atto643}$ at 643 nm is 150,000 $M^{-1}cm^{-1}$; and L = 1 cm. Based on these values, the average Atto643 labels per IgG molecule was calculated to be approximately 2.3.

Binding studies via GNP fluorescence quenching assay

To quantify the binding affinities between antibody and G13-His_6 of varying His_6 content (0–10%), HisAb-Atto643 with varied concentration from 0.1, 0.2, 0.4, 0.8, 1.6, 3.2 to 6.4 nM was mixed with 1 mol equivalent of G13-His_6 in PBS containing 1 mg/mL BSA and then incubated at room temperature for 30 min. Fluorescence emission spectra of Atto643-labeled proteins and their respective binding complexes were recorded using a Jobin Yvon FluoroMax-5 Spectrofluorometer. Samples were measured in a 0.7 mL precision quartz cuvette with a 10 mm path length. Fluorescence emission spectra were recorded over a range of 650 – 800 nm using a fixed excitation wavelength (λ_{EX}) of 630 nm. Fluorescence spectra of HisAb-Atto643 at the above concentrations without G13-His_6 conjugates were also recorded under identical conditions. The fluorescence spectra from 650 to 800 nm were integrated and used to calculate the quenching efficiency (QE) at each concentration (*C*). The apparent binding dissociation constants (K_d) were then determined by fitting the QE – *C* plots to the Hill's equation.[1]

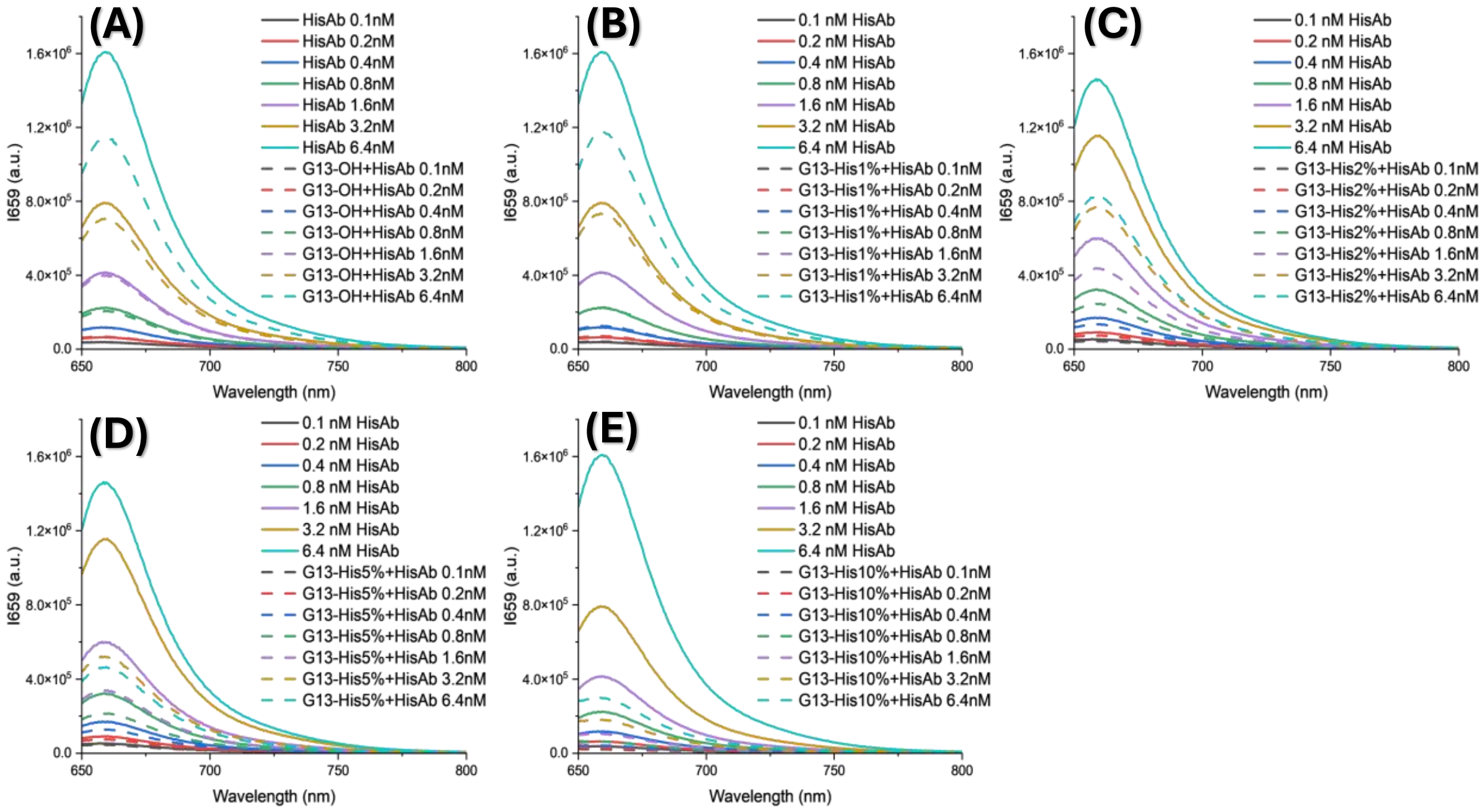

Figure S14. Fluorescence spectra of varying concentrations of HisAb-Atto643 in the absence (solid lines) and presence (broken lines) of 1 molar equivalent of G13-His_6 conjugates with His_6 densities of 0% (G13-OH, **A**), 1% (**B**), 2% (**C**), 5% (**D**), and 10% (**E**). The HisAb-Atto643 and G13-His_6 conjugate concentrations were both varied simultaneously from 0.1 to 6.4 nM, and their molar ratios were always fixed at 1: 1 in all fluorescence quenching experiments.

Reference

(1) Budhadev, D.; Poole, E.; Nehlmeier, I.; Liu, Y.; Hooper, J.; Kalverda, E.; Akshath, U. S.; Hondow, N.; Turnbull, W. B.; Pohlmann, S.; et al. Glycan-Gold Nanoparticles as Multifunctional Probes for Multivalent Lectin-Carbohydrate Binding: Implications for Blocking Virus Infection and Nanoparticle Assembly. *J Am Chem Soc* **2020**, *142* (42), 18022-18034. DOI: 10.1021/jacs.0c06793.

(2) Song, L.; Guo, Y.; Roebuck, D.; Chen, C.; Yang, M.; Yang, Z.; Sreedharan, S.; Glover, C.; Thomas, J.; Liu, D.; et al. Terminal PEGylated DNA-Gold Nanoparticle Conjugates Offering High Resistance to Nuclease Degradation and Efficient Intracellular Delivery of DNA Binding Agents. *ACS APPLIED MATERIALS & INTERFACES* **2015**, *7* (33), 18707-18716. DOI: 10.1021/acsami.5b05228.

(3) Hill, H. D.; Millstone, J. E.; Banholzer, M. J.; Mirkin, C. A. The Role Radius of Curvature Plays in Thiolated Oligonucleotide Loading on Gold Nanoparticles. *Acs Nano* **2009**, *3* (2), 418-424. DOI: 10.1021/nn800726e.

(4) Ning, X.; Budhadev, D.; Pollastri, S.; Nehlmeier, I.; Kempf, A.; Manfield, I.; Turnbull, W. B.; Pöhlmann, S.; Bernardi, A.; Li, X.; et al. Polyvalent Glycomimetic-Gold Nanoparticles Revealing Critical Roles of Glycan Display on Multivalent Lectin-Glycan Interaction Biophysics and Antiviral Properties. *JACS Au* **2024**, *4* (8), 3295-3309. DOI: 10.1021/jacsau.4c00610.
